# Transcriptome Profiling of Atlantic Salmon (*Salmo salar*) Parr With Higher and Lower Pathogen Loads Following *Piscirickettsia salmonis* Infection

**DOI:** 10.3389/fimmu.2021.789465

**Published:** 2021-12-31

**Authors:** Xi Xue, Albert Caballero-Solares, Jennifer R. Hall, Navaneethaiyer Umasuthan, Surendra Kumar, Eva Jakob, Stanko Skugor, Christopher Hawes, Javier Santander, Richard G. Taylor, Matthew L. Rise

**Affiliations:** ^1^ Department of Ocean Sciences, Memorial University of Newfoundland, St. John’s, NL, Canada; ^2^ Aquatic Research Cluster, CREAIT Network, Ocean Sciences Centre, Memorial University of Newfoundland, St. John’s, NL, Canada; ^3^ Cargill Innovation Centre - Colaco, Colaco, Chile; ^4^ Cargill Aqua Nutrition, Cargill, Sea Lice Research Center (SLRC), Sandnes, Norway; ^5^ Marine Microbial Pathogenesis and Vaccinology Lab, Department of Ocean Sciences, Memorial University of Newfoundland, St. John’s, NL, Canada; ^6^ Cargill Animal Nutrition and Health, Elk River, MN, United States

**Keywords:** Salmonid rickettsial septicaemia (SRS), EM-90, *Salmo salar*, microarray, immune response, biomarkers, freshwater, disease resistance

## Abstract

Salmonid rickettsial septicemia (SRS), caused by *Piscirickettsia salmonis*, is one of the most devastating diseases of salmonids. However, the transcriptomic responses of Atlantic salmon (*Salmon salar*) in freshwater to an EM-90-like isolate have not been explored. Here, we infected Atlantic salmon parr with an EM-90-like isolate and conducted time-course qPCR analyses of pathogen load and four biomarkers (*campb*, *hampa*, *il8a*, *tlr5a*) of innate immunity on the head kidney samples. Transcript expression of three of these genes (except *hampa*), as well as pathogen level, peaked at 21 days post-injection (DPI). Multivariate analyses of infected individuals at 21 DPI revealed two infection phenotypes [lower (L-SRS) and higher (H-SRS) infection level]. Five fish from each group (Control, L-SRS, and H-SRS) were selected for transcriptome profiling using a 44K salmonid microarray platform. We identified 1,636 and 3,076 differentially expressed probes (DEPs) in the L-SRS and H-SRS groups compared with the control group, respectively (FDR = 1%). Gene ontology term enrichment analyses of SRS-responsive genes revealed the activation of a large number of innate (e.g. “phagocytosis”, “defense response to bacterium”, “inflammatory response”) and adaptive (e.g. “regulation of T cell activation”, “antigen processing and presentation of exogenous antigen”) immune processes, while a small number of general physiological processes (e.g. “apoptotic process”, development and metabolism relevant) was enriched. Transcriptome results were confirmed by qPCR analyses of 42 microarray-identified transcripts. Furthermore, the comparison of individuals with differing levels of infection (H-SRS vs. L-SRS) generated insights into the biological processes possibly involved in disease resistance or susceptibility. This study demonstrated a low mortality (~30%) EM-90-like infection model and broadened the current understanding of molecular pathways underlying *P. salmonis*-triggered responses of Atlantic salmon, identifying biomarkers that may assist to diagnose and combat this pathogen.

## 1 Introduction

There is an excellent potential for the aquaculture industry to expand to fulfill the worldwide seafood demand for human consumption (1). Atlantic salmon (*Salmo salar*) is one of the most economically important species currently farmed in the marine finfish aquaculture sector (2). However, infectious diseases have resulted in substantial mortality and losses for Atlantic salmon aquaculture worldwide, affecting the growth and sustainability of the industry. Piscirickettsiosis or salmonid rickettsial septicemia (SRS) caused by the intracellular Gram-negative bacterial pathogen, *Piscirickettsia salmonis*, constitutes one of the main infectious diseases in salmonid aquaculture (3, 4). For example, in Chile, it was estimated by the National Fisheries and Aquaculture Service that about 50% of disease-causing mortalities in Atlantic salmon were attributed to piscirickettsiosis in 2020 (5). The economic losses caused by piscirickettsiosis to the Chilean aquaculture industry were approximately USD 700 million yearly (6, 7). Occurrences of this pathogen have also been reported in farmed salmonids in Norway, Scotland, Ireland and Canada (6, 8). *P. salmonis* causes systemic, chronic septicemia in fish, which is characterized by a variety of clinical signs, including a discolored and swollen head kidney, anemia, enlarged spleen, and skin and liver lesions (4, 7, 9). Although research progress has been made in several *in vitro* and *in vivo* models, the molecular mechanisms involved in *P. salmonis* pathogenesis are not entirely known (7, 9). Attempts to control this disease thus far have been unsuccessful due to the low efficacy of existing vaccines and antibiotic treatments (4, 9).

Previous transcriptomic analyses of Atlantic salmon challenged with live *P. salmonis* showed upregulation of genes involved in oxidative and inflammatory pathways (4, 10) as well as upregulation of genes related to cellular iron metabolism (4, 11). These studies revealed the activation of numerous immune responses upon *P. salmonis* infection. However, it remains unclear which of these immune responses are protective and what role is played by the host iron withholding response (also known as nutritional immunity), which aims to limit the access of bacteria to iron and heme, by promoting intracellular storage away from invading pathogens (12, 13). The key iron regulator Hepcidin exerted a protective effect against the extracellular *Flavobacterium columnare* in grass carp (*Ctenopharyngodon idella*) (14), while suppression of *hepcidin* was associated with protection in Atlantic salmon challenged by the facultative intracellular *Aeromonas salmonicida* bacterium (15). In contrast, a previous study with an iron chelator that successfully reduced the amount of available iron, had induction of *hepcidin-1* in multiple organs of Atlantic salmon undergoing *P. salmonis* infection that coincided with improved survival compared with the groups supplemented with iron (16). The study of Díaz et al. (12) also showed positive effects of the iron chelator deferoxamine mesylate in *P. salmonis* infected salmon SHK-1 cells which coincided with an increase in *hepcidin* expression. Possible host-pathogen interactions, such as the suppression of genes encoding pro-apoptotic proteins and induction of cell proliferation-related genes were also suggested in early studies (4, 10). The modulation of these host pathways may represent a mechanism used by *P. salmonis* to ensure the maintenance of host cells and to allow them to survive and replicate within host cells (10). In addition, this pathogen may inhibit the adaptive immune response in infected fish as a mechanism to evade host defense and promote replication and survival (4, 10, 17).

In Chile, the existence of two distinct *P. salmonis* genogroups, LF-89-like and EM-90-like strains, has been reported (6, 9, 18). Saavedra et al. (18) found that EM-90-like isolates, similar to LF-89-like ones, are highly prevalent and disseminated across Chilean marine farms. Studies revealed differences between these two genogroups in relation to geographic distribution, antibiotic susceptibility and host specificity (18). For example, *P. salmonis* field isolates showed a marked host preference with EM-90-like isolates exclusively recovered from farmed Atlantic salmon (18). Comparative pan-genome analysis of LF-89 and EM-90 strains has identified strain-specific virulence factors linked to adherence, colonization, invasion factors, and endotoxins (9). These genomic divergences may directly be associated with inter-genogroup differences in pathogenesis and host-pathogen interactions (9, 17, 19, 20). As a result, there has been a growing interest in studying the piscirickettsiosis resulting from EM-90-like isolates (6, 17, 19–21). Rozas-Serri et al. (17) evaluated the transcriptomic changes of post-smolt Atlantic salmon challenged with an EM-90-like *P. salmonis via* cohabitation in seawater. Fish infected with EM-90-like isolate showed extremely high mortality (100% in Trojans and 94.9% in cohabitant fish) (17, 19, 20). However, relatively small numbers of differentially expressed genes (298 in Trojans and 170 in cohabitant fish) were identified. Meza et al. (21) also analyzed the disease development of salmon after being challenged with an EM-90-like strain in seawater and resulted in 100% mortality in both Trojan and cohabitant fish. The transcriptomic responses of Atlantic salmon parr in freshwater to an EM-90-like isolate have not been explored. Although piscirickettsiosis has often been reported in seawater-reared salmonids, previous reports have documented the disease in coho salmon (*Oncorhynchus kisutch*) and rainbow trout (*O. mykiss*) farmed in freshwater (22, 23), and experimental infection trials have been successfully demonstrated in freshwater reared coho salmon, Atlantic salmon and rainbow trout (24–26).

In the present study, we aimed to explore the head kidney transcriptomic responses of the Atlantic salmon parr from a low mortality EM-90-like *P. salmonis* infection trial. A low mortality infection model is needed to fill the gaps in the current knowledge regarding piscirickettsiosis outbreaks involving EM-90-like strains; this model can also be beneficial in evaluating the performance of novel functional feeds designed to improve fish health and mitigating/reducing disease incidence in aquaculture. Samples collected from multiple time points post-infection (up to 42 days) were included to study the temporal patterns of pathogen level and host immune responses. All exposed fish in the present study received the same dose of *P. salmonis*; however, two infection phenotypes (higher and lower infection levels) at 21 days post-injection (DPI) were detected by multivariate analyses of pathogen load and levels of 4 anti-bacterial transcript biomarkers. A number of previous disease challenge studies in Atlantic salmon have shown that pathogen load can be used as a good indicator of disease resistance (11, 27–29). Dettleff et al. (27) found that the bacterial load of fish infected with *P. salmonis* was significantly lower in resistant individuals when compared with the susceptible ones; the resistant fish had increased expression of *c-type lysozyme* in the head kidney, while the susceptible fish demonstrated an exaggerated innate immune response. Therefore, a specific focus on the transcriptional differences between lower and higher infection individuals (i.e. L-SRS vs. H-SRS) was also attempted using the consortium for Genomic Research on All Salmonids Project (cGRASP)-designed Agilent 44K salmonid oligonucleotide microarray (30). This particular microarray platform has proven to be a useful and robust tool in detecting differentially expressed transcripts in several immune-related studies in Atlantic salmon and rainbow trout (31–37). We hypothesize that the exploration into transcriptomic differences between high and low infection individuals may provide insight into the molecular mechanisms associated with the ability to evade or clear *P. salmonis* infection as well as the dysregulations leading to susceptibility and more adverse outcomes. Finally, this study revealed candidate biomarker genes that could be valuable for future SRS-related research.

## 2 Materials and Methods

### 2.1 Experimental Animals

The *P. salmonis* disease challenge trial was conducted at the Cargill Innovation Center (Colaco, Chile). Atlantic salmon parr (64.2 ± 10.4 g mean initial weight ± SD) were randomly distributed into 10 challenge tanks (200 L) with 125 fish/tank. Prior to the study, 30 fish were screened for *Flavobacterium psychrophilum*, *P. salmonis*, *Renibacterium salmoninarum*, infectious pancreatic necrosis virus (IPNV) and infectious salmon anemia virus (ISAV) using real-time quantitative polymerase chain reaction (qPCR), and were negative for all of these pathogens (data not shown). Fish were kept in a flow-through freshwater system (~15.2°C and 24 h light). Oxygen in the tank was maintained at 92.3 ± 7.3% (mean ± SD) of saturation. Fish were held for 14 days prior to the start of the infection trial to acclimate to the tank conditions. A standard EWOS commercial diet (EWOS Micro 50) was supplied using automatic feeders over a period of 18 h per day (from 02:00 pm to 08:00 am) and fed to satiation. Fish were fasted 12 h before and after all experimental procedures (e.g. injection and sampling).

**Figure 1:**
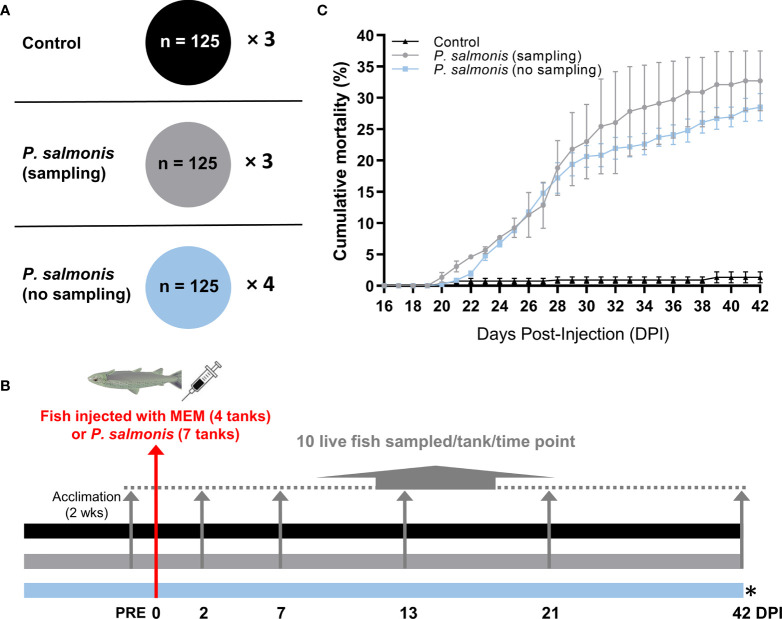
Overview of the infection trial, sampling and cumulative mortality. **(A)** The tank allocation and the number of fish for each treatment group [Control (MEM media injected), *P. salmonis-*injected (sampling), *P. salmonis*-injected (no sampling)]. **(B)** Timeline of the overall study with sampling regime showing the number of days post-infection (DPI). Four out of 7 tanks in the challenge group [i.e. *P. salmonis*-injected (no sampling)] were left untouched in order to monitor mortality. **(C)** Cumulative mortality in each treatment group. No statistical significance was found in the cumulative mortality by the end of the infection trial between *P. salmonis*-injected (sampling) and *P. salmonis*-injected (no sampling) groups (*p* > 0.05; Student’s *t* test).

An EM90-like strain of *P. salmonis* was purchased from Fundacion Fraunhofer Chile Research (Puerto Montt, Chile). The bacteria were grown on CHSE-214 cell cultures according to standard operating procedures of Fundacion Fraunhofer Chile Research, and the inoculum with the desired strength was prepared and transferred under chilled conditions within 1 h to the Cargill Innovation Center challenge facility on the day of the infection. The inoculum (i.e. suspension of CHSE-214 cells infected with *P. salmonis*) was checked for contaminations (i.e. IPNV, ISAV, *Piscine reovirus*, *R*. *salmoninarum*, *Flavobacterium* sp., *Aeromonas salmonicida*) using qPCR. The titer of the inoculum [i.e. median tissue culture infective dose (TCID_50_)] was determined on CHSE-214 cells using an endpoint dilution assay. All fish from the challenge group (7 tanks) were anesthetized using benzocaine (150 µL L^-1^ BZ-20^®^, Veterquímica S.A., Maipú, Santiago, Chile) and intraperitoneally (IP) injected with 0.1 mL of bacterial inoculum with a titer of 10^0.83^ TCID_50_/mL. The target titer of 10^1^ TCID_50_/mL (real titer 10^0.83^ TCID_50_/mL) was chosen based on results from previously conducted dose dilution trials (10^1^-10^4^ TCID_50_/mL) with the same bacterial isolate at the Cargill Innovation Center. The aim of this trial was to achieve a slow onset of mortality and to reach about 30-40% accumulative mortality over the duration of the trial to evaluate the pathological changes over time. Fish from the mock-injection control group (3 tanks) were anesthetized as above, and IP injected with 0.1 mL of the medium that was used to prepare the bacterial inoculum (i.e. minimum essential medium, MEM) (**Figures 1A, B**). Ten fish per tank [3 tanks from the challenge group (i.e. *P. salmonis*-injected) and 3 tanks from the control group (CON)] were euthanized using an overdose of benzocaine (300 µL L^-1^ BZ-20^®^) and dissected on the day before injection (PRE) of the remaining fish, as well as 2, 7, 13, 21 and 42 DPI. Head kidney samples were collected and stored in RNAlater (Ambion/Life Technologies, Austin, TX, USA) for 48 h at 4°C and transferred to -80°C until RNA extraction. Four out of 7 tanks in the challenge group were left untouched in order to monitor mortality (**Figures 1A, B**). All procedures in this study were conducted following the guidelines of the Canadian Council on Animal Care (38).

### 2.2 RNA Extraction, DNase Treatment, and Column Purification

RNAlater was removed prior to RNA extraction. All samples were lysed in TRIzol reagent (Invitrogen/Life Technologies, Carlsbad, CA, USA) using a tissue homogenizer (Precellys 24, Bertin Instruments, Montigny-le-Bretonneux, France), and subjected to RNA extraction according to the manufacturer’s instructions. Forty micrograms of each total RNA sample were treated with 6.8 Kunitz units of DNase I (QIAGEN, Mississauga, ON, Canada) to degrade residual genomic DNA, and then purified using the RNeasy Mini Kit (QIAGEN) following the manufacturer’s protocols. RNA integrity was verified by 1% agarose gel electrophoresis, and RNA purity was assessed by A260/280 and A260/230 ratios using NanoDrop spectrophotometry (Thermo Fisher, Mississauga, ON, Canada).

### 2.3 qPCR Analysis of Pathogen Load and Candidate Host Immune Biomarkers

For pathogen load assessment, specific primers and probe for the *P. salmonis 16S-23S ribosomal RNA internal transcribed spacer* (*ITS*; in-house developed by Cargill Innovation) were used for pathogen load detection, with Atlantic salmon *elongation factor 1 alpha 1* (*ef1a1*) (39) levels analyzed as an internal control (i.e. reference gene). Primer and probe sequences for these genes can be found in **Supplemental Table S1**. Pathogen load was assessed across all available samples (*n* = 337) in technical duplicates using a probe-based detection (i.e. TaqMan) kit AGPATH-ID ONE-STEP RT-PCR (Ambion/Life Technologies) and the ViiA7 Real-Time PCR system (Applied Biosystems/Life Technologies). The reaction mix contained 6.5 μL of 2X RT-PCR Buffer, 0.52 μL of 25X RT-PCR Enzyme Mix, 0.88 μL detection enhancer, 900 nM of forward primer, 600 nM of reverse primer, 225 nM of probe, 50 ng of RNA template, and DEPC-treated water to reach a final volume of 13 μL. The real-time PCR thermal cycling profile consisted of the reverse transcription step at 45°C for 10 min, the reverse transcriptase inactivation/initial denaturation step at 95°C for 10 min, and the amplification step of 45 cycles of 95°C for 15 s and 60°C for 45 s. Prior to pathogen load assessment, head kidney RNA templates from three infected animals were selected to test the performance and amplification efficiencies of primers, and determine the optimal input RNA quantity. Amplification efficiencies (40) of *ITS* and *ef1a1* for pathogen load assessment were determined from three standard curves performed using a 5-point 1:3 dilution series starting with 150 ng of input total RNA. The reported efficiencies are an average of the three values. The infection level assays for each sample were then measured using 50 ng of input RNA in each reaction. No-template and positive (RNAs from three highly infected fish) controls were included in each plate run. The relative quantity (RQ) of *P. salmonis ITS* was determined using the ViiA 7 Software Relative Quantification Study Application (Version 1.2.3) (Applied Biosystems/Life Technologies), with normalization to *ef1a1* transcript levels, and with amplification efficiencies incorporated. The sample with the lowest detectable expression level was set as the calibrator sample (i.e. assigned an RQ value = 1.0).

Following the bacterial screening, smaller sample sizes of the control fish (i.e. PRE and time-match controls) together with all available fish from the *P. salmonis*-injected group (except for 2 DPI) (total *n* = 192) were further subjected to qPCR analyses of 4 well-known antibacterial biomarkers [*campb* (17, 34, 41–43), *hampa* (11, 31, 41), *il8a* (10, 19, 41), and *tlr5a* (19, 34)] to assess the infection and host immune response dynamics. The qPCR analysis for these genes was conducted on a ViiA7 384 well platform (Applied Biosystems/Life Technologies), and the methods (e.g. cDNA synthesis, primer quality control, and PCR program) for these assays are described in Section 2.5. Transcript levels of these four biomarkers were normalized to transcript levels of two endogenous control genes. To select these control genes, the expression of 6 candidate normalizers [i.e. *60S ribosomal protein 32* (*rpl32*; BT043656), *β-actin* (*actb*; BG933897), *ef1a1* (AF321836), *ef1a2* (BT058669), *eukaryotic translation initiation factor 3 subunit D* (*eif3d*; GE777139), and *polyadenylate-binding protein 1* (*pabpc1*; EG908498)] was measured in 48 out of 192 individuals (i.e. 25%) involved in this screen step and then analyzed using *geNorm* (44). Using this software, *eif3d* (*geNorm* M = 0.217) and *rpl32* (*geNorm* M = 0.211) were determined to be the most stable across infection levels and time points. The expression levels (RQ) of these genes were analyzed using a two-way ANOVA test, followed by Sidak multiple comparisons *post hoc* test in order to identify significant differences between *P. salmonis* injected and control groups at each time point, and within one treatment group at multiple time points. The normality of the qPCR data (i.e., RQ values) was analyzed using the Shapiro-Wilk and Kolmogorov-Smirnov normality tests. All of the statistical tests above were performed using Prism v7.0 (GraphPad Software Inc., La Jolla, CA, USA). Differences were considered statistically significant when *p* < 0.05.

### 2.4 Transcriptome Profiling by 44K Microarray

#### 2.4.1 Selection of Time Point and Individuals

**Figure 2:**
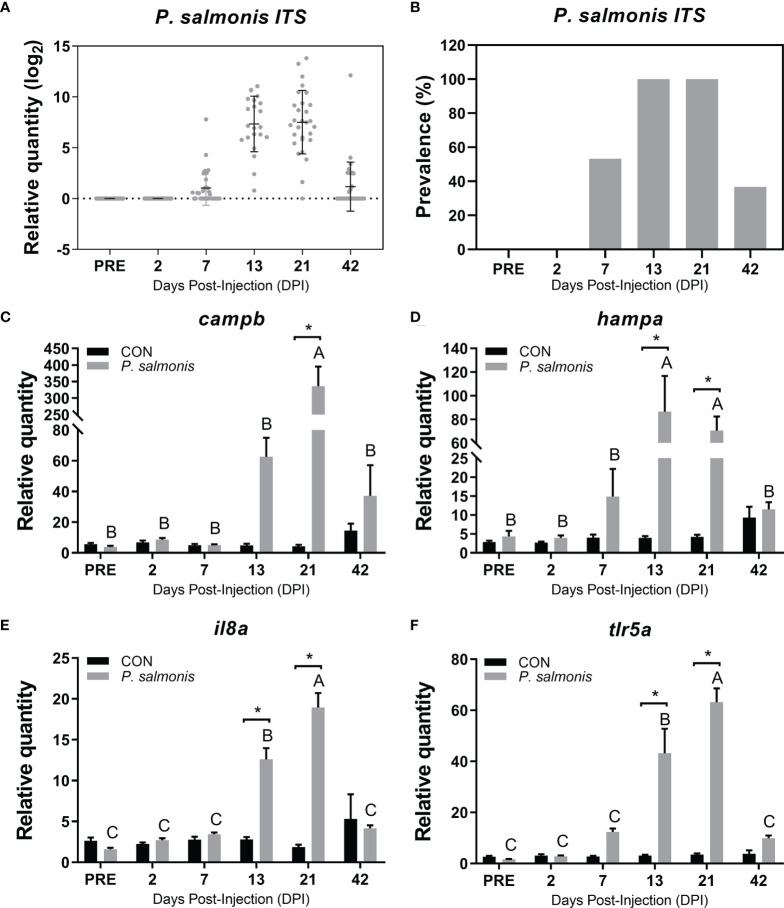
Detection of *P. salmonis* transcript and evaluation of candidate immune biomarkers in the head kidney of Atlantic salmon across different time points. **(A)** Transcript levels (i.e. relative quantity) of *P. salmonis 16S-23S ribosomal RNA internal transcribed spacer* (*ITS*) in the head kidney of Atlantic salmon injected with the same dose of bacterial inoculum, as determined by TaqMan assays across all sampling time points. **(B)** The relative prevalence of *P. salmonis* positive (i.e. detection of *P. salmonis ITS*) in fish that were injected with bacterial inoculum. **(C–F)** qPCR analysis of host immune biomarkers (*campb*, *hampa*, *il8a*, *tlr5a*) in both control (CON) and *P. salmonis* injected fish. Data are presented as mean ± SE. An asterisk indicates a significant difference between time-matched *P. salmonis* injected and control groups. Different letters represent the significant differences within an injection group over time. All data presented in the figure are provided in **Supplemental Table S2**.

The RQ of *P. salmonis ITS* across the infection course peaked at 21 DPI, with all exposed fish testing positive (**Figures 2A, B**). Complete results of *P. salmonis ITS* expression levels in all samples can be seen in **Supplemental Table S2**. Similar to *P. salmonis ITS* levels, the transcript expression of *campb*, *il8a* and *tlr5a* also peaked at 21 DPI and was significantly higher compared with the time-matched control fish (**Figure 2**). Therefore, we decided to study the transcriptomic responses of Atlantic salmon to *P. salmonis* at the peak of infection and presumed peak of most immune activities (i.e. 21 DPI). In addition, multivariate statistical analyses (i.e. principal component analysis and hierarchical clustering) were performed using PRIMER (Version 6.1.15; PRIMER-E Ltd., Ivybridge, UK) to identify different infection phenotypes. Within infected fish, there were two infection level phenotypes identified at 21 DPI (except fish C7-8): one group of fish with higher transcript levels of *P. salmonis ITS* and the antibacterial biomarkers (H-SRS) and another group of fish with lower transcript levels of *P. salmonis ITS* and the antibacterial biomarkers (L-SRS) (**Supplemental Figures S1A, B**). The level of *P. salmonis ITS* in fish classified as H-SRS was significantly higher (19.1-fold) than that of L-SRS fish (**Supplemental Figure S1C**). Five individual fish from each infection phenotype (L-SRS and H-SRS) that were more closely clustered based on multivariate analysis were included in the microarray analysis along with five fish from the control group (i.e. CON) (**Supplemental Figure S1D**).

#### 2.4.2 Microarray Hybridization and Data Acquisition

The cGRASP-designed Agilent 4×44K salmonid oligonucleotide microarray (30, 32, 45) was used to evaluate the impact of *P. salmonis* infection on the Atlantic salmon head kidney transcriptome at 21 DPI. With a common reference design, 15 arrays were used in this study, with one array per individual fish. The microarray experiment was performed as previously described (31, 33, 45, 46). Briefly, anti-sense amplified RNA (aRNA) was *in vitro* transcribed from 1 µg of each RNA template using the Amino Allyl MessageAmp II aRNA Amplification kit (Ambion/Life Technologies), following the manufacturer’s instructions. For each fish, 20 μg of aRNA were precipitated overnight following standard molecular biology procedures and re-suspended in 9 μl of the manufacturer’s coupling buffer. To generate the common reference pool, 5 μg of aRNA from each of the fifteen samples were pooled, and divided into 20 μg aliquots, which were precipitated and re-suspended as above. Resulting aRNA was labelled with either Cy3 (for the common reference) or Cy5 (for the experimental individuals) fluor (GE HealthCare, Mississauga, ON, Canada) through a dye-coupling reaction, following the manufacturer’s instructions. Equal quantities (825 ng) of each labelled aRNA from one experimental sample and the common reference were pooled, fragmented following the manufacturer’s instructions and co-hybridized to a cGRASP-designed Agilent 44K salmonid oligonucleotide microarray (GEO accession # GPL11299) (30) as per the manufacturer’s instructions (Agilent, Mississauga, ON, Canada). The arrays were hybridized at 65°C for 17 h with 10 rpm rotation in an Agilent hybridization oven. The array slides were washed immediately following hybridization as per the manufacturer’s instructions. Slides were dried by centrifuging at 200 × g for 5 min at room temperature prior to scanning.

Each microarray was scanned at 5 µm resolution and 90% of laser power using a ScanArray Gx Plus scanner and ScanArray Express software (v4.0; Perkin Elmer, Woodbridge, ON, Canada) with photomultiplier tube (PMT) set to balance fluorescence signal between channels. The resulting TIFF images containing raw microarray data were extracted using Imagene (v9.0; BioDiscovery Inc., El Segundo, CA). Background correction, data transformation (log_2_), print-tip Loess normalization, and removal of low-quality/flagged spots were performed using R and the Bioconductor package *mArray* (45–47). After spot quality filtering, probes absent in more than 25% of the arrays (i.e. 4 arrays out of 15) were discarded, resulting in a final list of 20,701 probes for statistical analyses. All microarray data have been submitted to Gene Expression Omnibus (GEO) under the accession GSE178327.

#### 2.4.3 Differential Expression Analysis and Probe Annotation

Prior to statistical analyses, missing data points for the 20,701 probes were imputed using the EM_array method from LSimpute (48, 49). The differentially expressed probes (DEPs) among groups (i.e. L-SRS vs. CON; H-SRS vs. CON; H-SRS vs. L-SRS) were determined using Significance Analysis of Microarrays (SAM) (50). SAM was conducted at a false discovery rate (FDR) cutoff of 1% to focus on the most significant DEPs, using the Bioconductor package *siggenes* (51). The resulting gene lists were annotated using the probe sequences (60mer) for gene identification by BLASTn against the NCBI nr/nt databases for both Atlantic salmon and rainbow trout. Stringent filtering criteria (only 2 allowed mismatches with un-gapped alignment option) were applied for the 60mer probe BLASTn hits. The contiguous sequences (contigs) from which the microarray probes were designed were also used for gene identification by BLASTx against the NCBI Swiss-Prot database (April 2019 version). If there were no or non-informative BLASTn results for a given probe, then the best BLASTx hit that had an expect (E) value <10^-5^ and an informatively named protein hit was reported. The gene symbols for probes were assigned from HUGO Gene Nomenclature Committee (HGNC; https://www.genenames.org/) and/or GeneCards (https://www.genecards.org/) databases. Using R and *gplots* package, a hierarchically clustered heat map was constructed with microarray normalized log_2_ ratios (Cy5/Cy3 ratios) of all identified DEPs using Euclidean distance and Ward’s agglomerative linkage method (ward.D2).

#### 2.4.4 GO Term Enrichment Test and Visualization of GO Term Networks

GO term enrichment tests were performed on lists of DEPs (i.e. 166 L-SRS responsive only, 1470 overlap, and 1606 H-SRS responsive only) using ClueGO (52) and CluePedia plugins in Cytoscape (v3.7.0) (53). The biological process (BP) GO database (20.12.2019) was used. The enrichment analysis was performed using a right-sided hypergeometric test after its adjustment by the Bonferroni-Hochberg procedure with a p-value set at 0.01 (i.e. FDR = 1%). GO term fusion strategy and a Kappa-statistics score of 0.5 were employed to integrate GO categories, minimize the complexity, and create a functionally organized GO cluster network. For each cluster (reflected by a different color), a GO term with the lowest p-value was selected as the leading term in each functional group (i.e. highly connected terms within the GO network). As previously described in Eslamloo et al. (31), the enriched GO terms were classified using Gene Ontology Browser (http://www.informatics.jax.org) into 6 functional themes: (1) “adaptive immune response”; (2) “immune response”; (3) “response to stress”; (4) “development”; (5) “metabolic process”; and (6) “cellular process, localisation, and structure”. The enriched GO terms for DEPs responsive to L-SRS only were classified into three themes (“adaptive immune response”, “immune response”, and “cellular process, localization, and structure”). The GO terms were classified based on the biological process to which they were related and/or their parent terms (especially for highly specific terms). Finally, a hierarchically clustered heat map was constructed based on probes having the associated GO term “adaptive immune response” using Genesis (54); all data were median-centred and clustered using Pearson correlation and complete linkage hierarchical clustering as in Rise et al. (55).

### 2.5 qPCR Confirmation of Microarray-Identified DEPs

In total, 42 candidate *P. salmonis-*responsive biomarkers were selected for qPCR analysis. In addition to samples chosen for the microarray study, the remaining samples from both infection level groups (see *Selection of Time Point and Individuals* and **Supplemental Figure S1** for sample classification) at 21 DPI (total *n* = 37; 11 L-SRS, 17 H-SRS, and 9 CON) were also included in the qPCR experiment.

First-strand cDNA templates for qPCR were synthesized in 20 μL reactions from 1 µg of DNaseI-treated, column-purified total RNA using random primers (250 ng; Invitrogen/Life Technologies), dNTPs (0.5 mM final concentration; Invitrogen/Life Technologies) and M-MLV reverse transcriptase (200 U; Invitrogen/Life Technologies) with the manufacturer’s first strand buffer (1× final concentration) and DTT (10 mM final concentration) at 37°C for 50 min. PCR amplifications were performed in 13 μL reactions using 1× Power SYBR Green PCR Master Mix (Applied Biosystems/Life Technologies), 50 nM of both the forward and reverse primers, and the indicated cDNA quantity (see below). The real-time analysis program consisted of 1 cycle of 50°C for 2 min, 1 cycle of 95°C for 10 min and 40 cycles of 95°C for 15 sec and 60°C for 1 min, with fluorescence detection at the end of each 60°C step.

The sequences of all primer pairs used in qPCR analyses are presented in **Supplemental Table S1**. Each primer pair was quality tested using the QuantStudio 6 Flex Real-Time PCR System (384-well format) (Applied Biosystems/Life Technologies). Quality testing ensured that a single product was amplified (dissociation curve analysis) and that there was no primer-dimer present in the no-template control. Amplification efficiencies (40) were calculated using two cDNA templates that were pooled post-cDNA synthesis: one pool of 5 CON samples and one pool of 5 H-SRS samples. Standard curves were generated using a 5-point 1:3 dilution series starting with cDNA representing 10 ng of input total RNA. The reported efficiencies (**Supplemental Table S1**) are an average of the two values.

After completing the primer quality control tests, qPCR analyses of transcript expression levels of the target genes were performed using the QuantStudio 6 Flex Real-Time PCR System (Applied Biosystems/Life Technologies). Diluted cDNAs corresponding to 5 ng of input RNA were used as templates in the PCR reactions. The C_T_ values of the GOIs and references genes were determined using the QuantStudio Real Time PCR Software Relative Quantification Study Application (Version 1.3) (Applied Biosystems/Life Technologies). The RQ of each transcript was determined using a qBase relative quantification framework (56, 57), with normalization to both *rpl32* and *eif3d* (see *qPCR Analysis of Pathogen Load and Candidate Host Immune Biomarkers* for normalizer gene testing), and with amplification efficiencies incorporated. The RQs of each transcript were calibrated against the control group.

All qPCR data (i.e. RQ values) were subjected to the Grubbs’ test to identify potential outliers, and log_2_-transformed to meet the normality assumption. The normality of the qPCR data (i.e., log2 RQ values) was analyzed using the Shapiro-Wilk and Kolmogorov-Smirnov normality tests. In total, 19 RQ values were determined to be statistical outliers in the entire dataset (i.e., out of 1554 RQ values), and were excluded from the study. Fold-change values derived from microarray and qPCR were log_2_-transformed and analyzed for correlation *via* linear regression as performed in previous studies (33, 46, 58). A significant correlation (*p* < 0.05) between both datasets was considered as confirmation of the microarray results. Transcript expression differences between groups were analyzed using a one-way ANOVA followed by Tukey’s *post hoc* test for multiple comparisons at the 5% level of significance. Furthermore, the relationships between the expression of qPCR-studied transcripts and *P. salmonis* infection level were assessed using linear correlation analysis. Control fish were excluded from this analysis as all of these fish tested negative for *P. salmonis* (i.e. no RQ available). All of the statistical tests above were performed using Prism v7.0 (GraphPad Software Inc., La Jolla, CA, USA).

## 3 Results

### 3.1 Cumulative Mortality

The cumulative mortality of fish infected with an EM-90-like isolate (sampling and non-sampling groups), as well as non-infected controls, are shown in **Figure 1C**. The mortality rate in the sampling group does not consider fish that were removed for sample collection. The first mortalities in both *P. salmonis*-injected groups were registered at 20 DPI. The cumulative mortality of the *P. salmonis*-injected (sampling) group by the end of the infection trial reached 32.7%, which was not significantly different from the *P. salmonis*-injected (no sampling) group (i.e. 28.5%).

### 3.2 qPCR Analysis of Pathogen Load and Candidate Host Immune Biomarkers

The *P. salmonis ITS* transcript level was assessed using a Taqman assay. All head kidney samples for the PRE and 2 DPI were negative for the *ITS* transcript (**Figures 2A, B**). The infection prevalence of *P. salmonis* (detectable pathogen *ITS*) reached 100% at both 13 and 21 DPI, and the mean *ITS* expression level peaked at 21 DPI based on all available samples tested.

Four well-known antibacterial biomarker transcripts (*campb*, *hampa*, *il8a* and *tlr5a*) were analyzed *via* qPCR to assess the infection and host immune response dynamics (**Figures 2C–F**). The highest expression level of *campb*, *il8a* and *tlr5a* in response to *P. salmonis* infection was observed at 21 DPI, while the expression of *hampa* in the *P. salmonis-*injected group peaked at 13 DPI. All four genes assayed in the current study were significantly up-regulated in the *P. salmonis-*injected group at both 13 and 21 DPIs compared with time-matched mock-injection controls, except for *campb*, which was only significant at 21 DPI. Further multivariate statistical analyses of the infection level and the expression of 4 antibacterial biomarkers of individuals collected at 21 DPI revealed infection and immune response phenotypes (i.e. L-SRS and H-SRS), with the exception of fish C7-8 which did not group with either phenotype (**Supplemental Figures S1A, B**). Complete qPCR results for *P. salmonis ITS* level and these four antibacterial biomarkers can be found in **Supplemental Table S2**.

### 3.3 Global Transcriptomic Expression in Salmon Head Kidney

**Figure 3:**
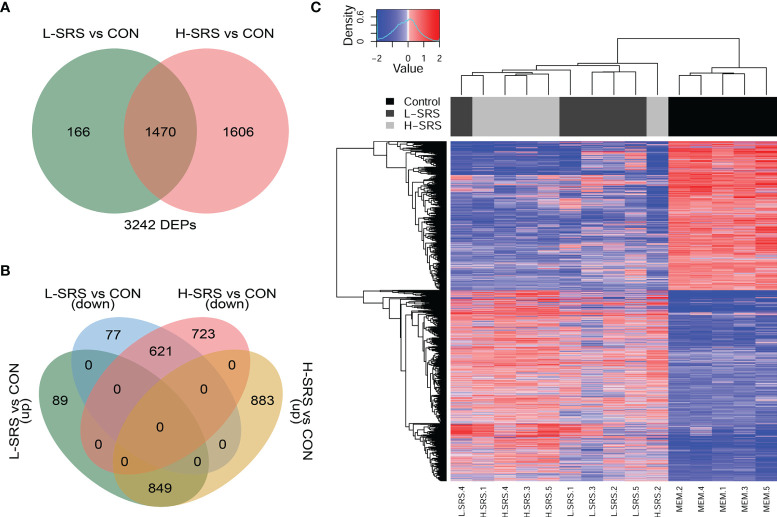
Overview of the head kidney transcriptomic responses of Atlantic salmon with higher (H-SRS) and lower (L-SRS) levels of *P. salmonis* compared with the control group at 21 days post-injection (DPI). Differentially expressed probes (DEP) were identified by Significance Analysis of Microarray (FDR = 1%). **(A)** Venn diagram illustrates the total number of DEPs in L-SRS or H-SRS groups in comparison to the control fish (CON). **(B)** Venn diagram shows the corresponding number of DEPs that were up- or down-regulated in L-SRS or H-SRS groups in comparison to the control fish (CON). **(C)** Hierarchical clustering analyses of samples based on all *P. salmonis* responsive probes (n = 3242).

In this study, we used the cGRASP-designed Agilent 44K microarray platform (30) to explore the transcriptomic response of Atlantic salmon head kidney to *P. salmonis* at 21 DPI. Fish showing a lower (L-SRS) or a higher (H-SRS) level of the *P. salmonis* infection were selected for comparison of their transcriptome profiles with the mock-injection control group (CON). The overall results of the microarray study, including the number of genes identified in each comparison, are summarised in **Figures 3A, B**. In total, 3242 significantly differentially expressed probes (DEPs) were identified in the *P. salmonis*-injected groups using SAM analyses (FDR = 1%) compared to the control (CON). However, SAM analysis failed to detect any significant DEPs when comparing H-SRS and L-SRS groups directly. The complete gene lists, including gene names and symbols, are shown in **Supplemental Table S3**. As illustrated by the Venn diagrams (**Figures 3A, B**), the two significant comparisons in the present study shared 1470 DEPs (849 up- and 621 down-regulated). Among these DEPs, the direction of gene expression regulation by *P. salmonis* infection was all the same among H-SRS and L-SRS when compared with the control group; however, they often differed in the degree of regulation (i.e. fold-changes) with the H-SRS group generally showing higher fold-changes (**Figure 3C**). In addition, there was a larger number of SRS-responsive probes in the H-SRS group compared with the L-SRS group (i.e. 3076 vs. 1636), suggesting a higher level of *P. salmonis* infection was associated with a more pronounced gene expression response. Using all identified DEPs in hierarchical analyses, all mock-injection control samples clustered in a separate branch from all infected samples; however, within each infected group (i.e. L-SRS or H-SRS), only 4 out of 5 samples were clustered together (**Figure 3C**).

### 3.4 Insight Into Biological Pathways Regulated by *P. salmonis* (L-SRS vs H-SRS)

**Figure 4:**
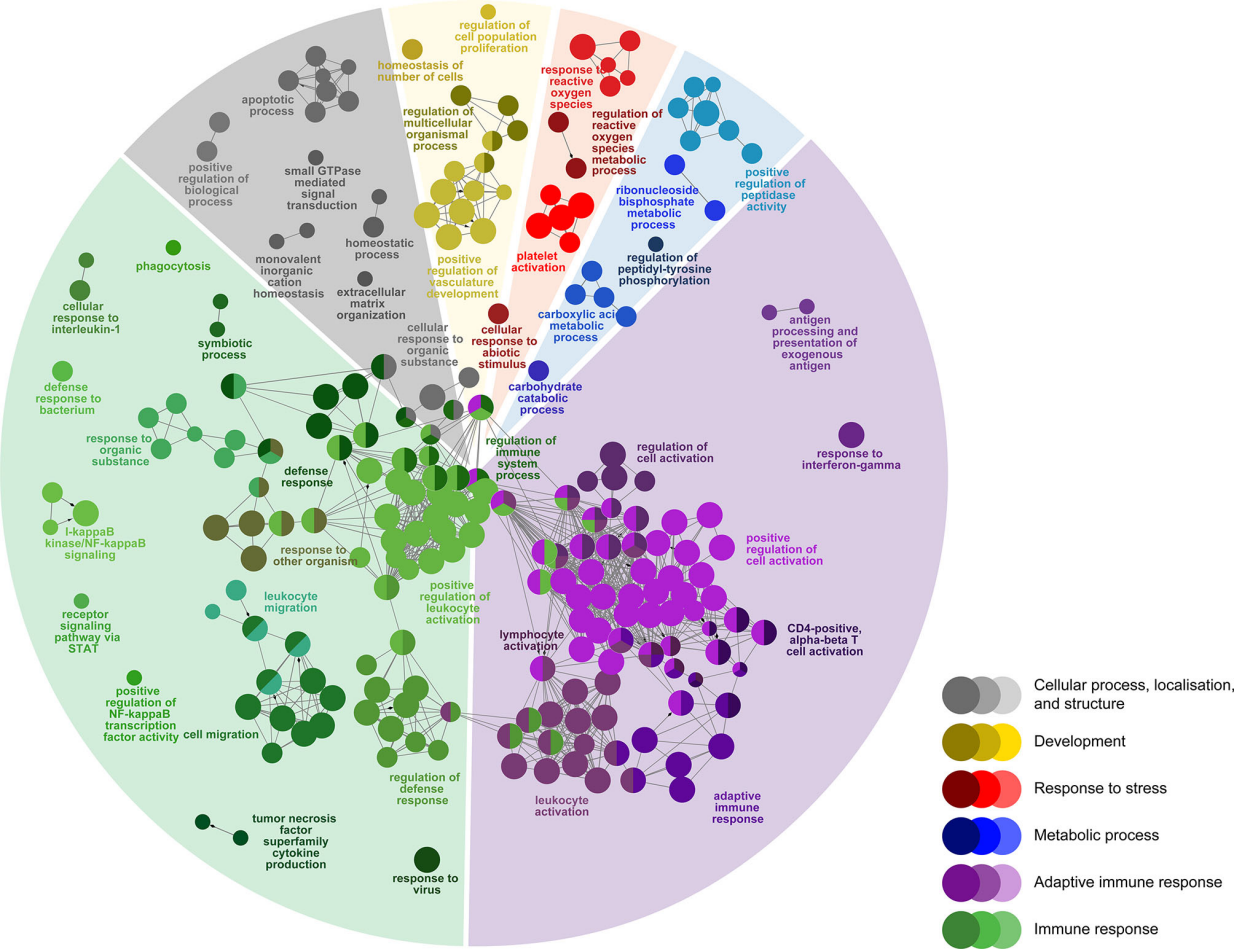
Enriched biological processes GO term networks of SRS-responsive probes (n = 1470) in the head kidney of Atlantic salmon at 21 days post-injection (DPI) shared by both H-SRS and L-SRS groups. Nodes represent significantly enriched GO terms [right-sided hypergeometric test, p-values (p < 0.01) corrected by Benjamini-Hochberg]. GO terms are grouped by functional theme and arranged to fit the sectors of a pie chart representing the proportion of GO terms in each functional theme. Nodes are colored according to the functional theme to which they were assigned. Grey lines in between GO terms indicate high connectivity (kappa coefficient > 0.5). Lines with arrows indicate the direction of positive regulation between GO terms, and lines with diamonds indicate the direction of regulation between GO terms.

To identify host molecular pathways regulated by *P. salmonis* infection, we compared the GO Biological Process (BP) term compositions of SRS-responsive transcript lists to that of the whole array using a Fisher’s exact test in ClueGO (FDR = 1%). First, we identified 292 BP terms enriched in SRS-responsive transcripts (1,470 DEPs) overlapping both infection groups (L-SRS and H-SRS) (**Figure 4**; **Supplemental Table S4**). The BP terms enriched by SRS (**Figure 4**) were associated with adaptive immune response (38.0%), immune response (36.3%), cellular process, localisation, and structure (10.3%), development (5.8%), metabolic process (5.1%), and response to stress (4.5%). A large number of BP terms involved in adaptive immunity were enriched by *P. salmonis* infection in Atlantic salmon. These included many terms related to lymphocyte differentiation and activation (e.g. “B cell activation”, “regulation of T cell activation”, “lymphocyte proliferation”, “immunoglobulin mediated immune response”, “response to interferon-gamma”), and some terms related to antigen presentation processes (e.g. “antigen processing and presentation of exogenous antigen”) (**Figure 4**; **Supplemental Table S4**). A total of 81 DEPs contributing to the enriched GO term, “adaptive immune response”, were used in constructing a heatmap to reveal the transcript profile of adaptive immunity-related genes (**Supplemental Figure S2**). Among these genes, 66 DEPs (e.g. *tap1*, *tap2*, *ifng*, *irf1*, *cd209a*, *bcl10*, *ada*) were up-regulated and 15 DEPs (e.g. *il7r*, *cd5*, *foxp3*) were down-regulated by *P. salmonis* infection (**Supplemental Figure S2**). A diverse and large group of BP terms related to the innate immune response (e.g. “phagocytosis”, “defense response to bacterium”, “response to interleukin-1”, “response to virus”, “chemotaxis”, “leukocyte migration”, “inflammatory response”) and regulation of immune response (e.g. “regulation of I-kappaB kinase/NF-kappaB signalling”, “regulation of defense response”, “regulation of leukocyte migration”) were activated in response to SRS infection. In addition, pathways related to various cellular processes (e.g. “positive regulation of programmed cell death”, “positive regulation of peptidase activity”, “homeostatic process”) were regulated in *P. salmonis*-infected salmon (**Figure 4**; **Supplemental Table S4**). Lastly, SRS affected head kidney genes related to development (e.g. “positive regulation of vasculature development”), metabolic process (e.g. “carboxylic acid metabolic process”), and response to stress (e.g. “regulation of reactive oxygen species metabolic process”) (**Figure 4**; **Supplemental Table S4**).

**Figure 5:**
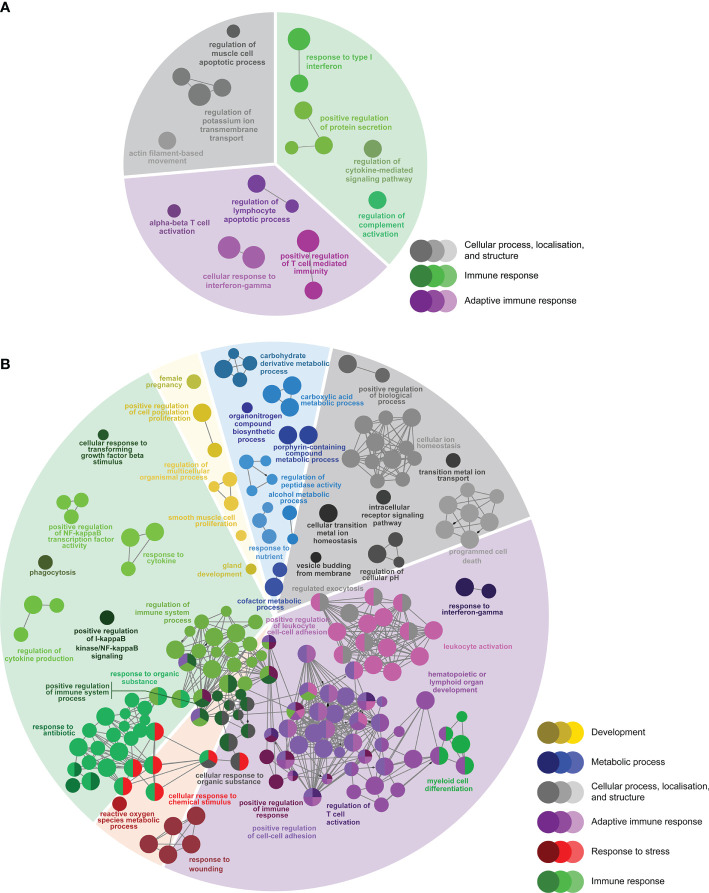
Enriched biological processes GO term networks of **(A)** SRS-responsive probes only identified in L-SRS group (n = 166) and **(B)** SRS-responsive probes only identified in H-SRS group (n = 1606) in the head kidney of Atlantic salmon at 21 days post-injection (DPI). Nodes represent significantly enriched GO terms [right-sided hypergeometric test, p-values corrected by Benjamini-Hochberg of 0.01 (i.e. FDR = 1%)]. GO terms are grouped by functional theme and arranged to fit the sectors of a pie chart representing the proportion of GO terms in each functional theme. Nodes are colored according to the functional theme to which they were assigned. Grey lines in between GO terms indicate high connectivity (kappa coefficient > 0.5). Lines with arrows indicate the direction of positive regulation between GO terms, and lines with diamonds indicate the direction of regulation between GO terms.

There were 166 DEPs only identified in the L-SRS group (L-SRS vs. Control) associated with several immune-related BP terms (73.6%), including “response to type I interferon”, “regulation of cytokine-mediated signaling pathway”, and “alpha-beta T cell activation”. In addition, many of these DEPs were involved in cellular process, localisation, and structure (26.3%), such as “regulation of muscle cell apoptotic process” and “actin filament-based movement” (**Figure 5A**; **Supplemental Table S5**). A much larger set of DEPs (n = 1606) was identified only in the H-SRS group (H-SRS vs. Control), and their over-represented BP terms (n = 269; **Figure 5B**; **Supplemental Table S6**) were similar in number and diversity to the enriched terms associated with the overlapping SRS-responsive DEPs. Briefly, 37.5% of the enriched BP terms were linked with the adaptive immune response (e.g. “regulation of T cell activation”, “response to interferon-gamma”, “B cell differentiation”, “hematopoietic or lymphoid organ development”), and 31.2% were associated with the immune response (e.g. “myeloid cell differentiation”, “regulation of cytokine production”) (**Figure 5B**; **Supplemental Table S6**). The enriched BPs in the transcript list only identified in the H-SRS group also included terms related to cellular process, localisation, and structure (15.6%; e.g. “cellular transition metal ion homeostasis”, “programmed cell death”, “vesicle budding from membrane”), metabolic process (7.8%; e.g. “carbohydrate derivative metabolic process”), response to stress (4.8%; e.g. “reactive oxygen species metabolic process”), and development (3.0%; e.g. “regulation of multicellular organismal process”) (**Figure 5B**; **Supplemental Table S6**).

### 3.5 qPCR Confirmation

**Figure 6:**
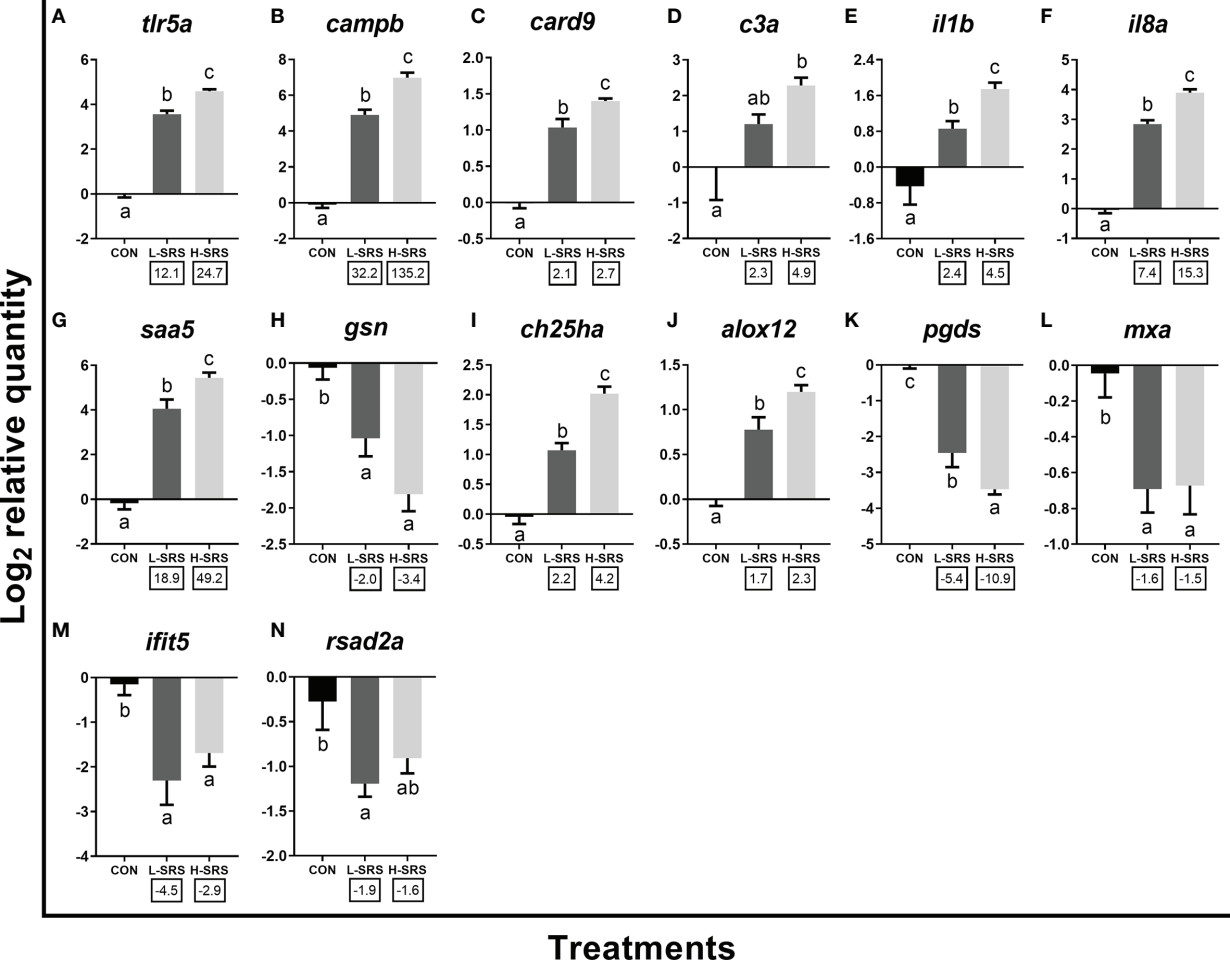
qPCR analyses of *P. salmonis*-responsive genes involved in the innate immune response. Fish with lower (L-SRS; n = 11) and higher (H-SRS; n = 17) level of *P. salmonis* infection at 21 days post-injection (DPI) as well as time-matched control (CON; n = 9) were used for qPCR confirmation. Average log_2_ RQs with SE bars are plotted. Different letters represent the significant differences among treatments (*p* < 0.05) with fold-change relative to control indicated below the x-axis. For qPCR fold-change calculation, overall fold up-regulation was calculated as 2^A-B^, where A is the mean of log_2_ RQ from the L-SRS or H-SRS groups, and B is the mean of log_2_ RQ from the CON group. For down-regulated transcripts, fold-change values were further inverted (-1/fold-change). **(A)** *toll-like receptor 5a*; **(B)** *cathelicidin antimicrobial peptide b*; **(C)** *caspase recruitment domain-containing protein 9*; **(D)** *complement c3a*; **(E)** *interleukin 1 beta*; **(F)** *interleukin 8a* (alias *C-X-C motif chemokine ligand 8a*); **(G)** *serum amyloid A-5 protein*; **(H)** *gelsolin precursor*; **(I)** *cholesterol 25-hydroxylase a*; **(J)** *arachidonate 12-lipoxygenase, 12S-type*; **(K)** *lipocalin-type prostaglandin D synthase*; **(L)** *interferon-induced GTP-binding protein Mx a*; **(M)** *interferon-induced protein with tetratricopeptide repeats 5*; **(N)** *radical S-adenosyl methionine domain-containing protein 2a*.

**Figure 7:**
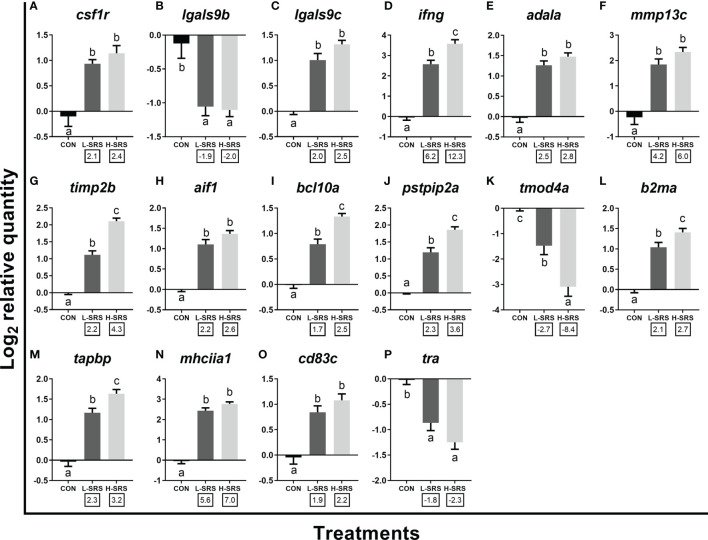
qPCR analysis of *P. salmonis*-responsive genes involved in cellular immunity (e.g. leukocyte activation and function). Fish with lower (L-SRS; n = 11) and higher (H-SRS; n = 17) level of *P. salmonis* infection at 21 days post-injection (DPI) as well as time-matched control (CON; n = 9) were used for qPCR confirmation. Average log_2_ RQs with SE bars are plotted. Different letters represent the significant differences among treatments (*p* < 0.05) with fold-change relative to control indicated below the x-axis. For qPCR fold-change calculation, overall fold up-regulation was calculated as 2^A-B^, where A is the mean of log_2_ RQ from the L-SRS or H-SRS groups, and B is the mean of log_2_ RQ from the CON group. For down-regulated transcripts, fold-change values were further inverted (-1/fold-change). **(A)** *macrophage colony-stimulating factor 1 receptor*; **(B)** *galectin 9b*; **(C)** *galectin 9c*; **(D)** *interferon gamma;* **(E)** *adenosine deaminase-like a*; **(F)** *matrix metalloproteinase 13c*; **(G)** *metalloproteinase inhibitor 2b*; **(H)** *allograft inflammatory factor 1*; **(I)** *B-cell lymphoma/leukemia 10a*; **(J)** *proline-serine-threonine phosphatase-interacting protein 2a*; **(K)** *tropomodulin-4a*; **(L)** *beta-2-microglobulin precursor a*; **(M)** *tapasin*; **(N)** *MHC class II antigen alpha chain 1*; **(O)** *cd83 antigen c*; **(P)** *T cell receptor alpha*.

**Figure 8:**
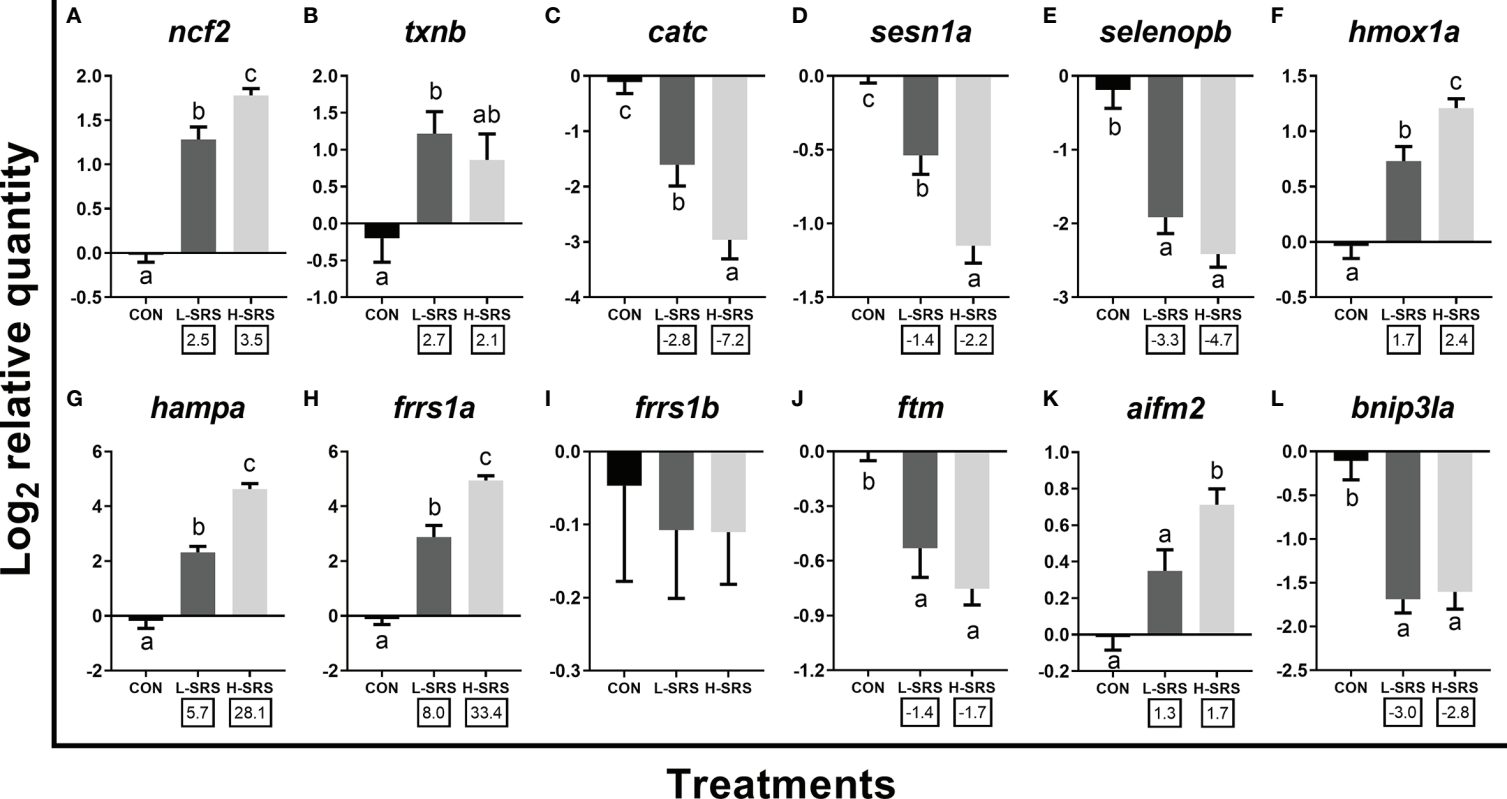
qPCR analysis of *P. salmonis*-responsive genes involved in redox homeostasis and other cellular processes. Fish with lower (L-SRS; n = 10) and higher (H-SRS; n = 16) level of *P. salmonis* infection at 21 days post-injection (DPI) as well as time-matched control (CON; n = 8) were used for qPCR confirmation. Average log_2_ RQs with SE bars are plotted. Different letters represent the significant differences among treatments (*p* < 0.05) with fold-change relative to control indicated below the x-axis. For qPCR fold-change calculation, overall fold up-regulation was calculated as 2^A-B^, where A is the mean of log_2_ RQ from the L-SRS or H-SRS groups, and B is the mean of log_2_ RQ from the CON group. For down-regulated transcripts, fold-change values were further inverted (-1/fold-change). **(A)** *neutrophil cytosol factor 2*; **(B)** *thioredoxin b*; **(C)** *catalase c*; **(D)** *sestrin-1a*; **(E)** *selenoprotein p b*; **(F)** *heme oxygenase 1a*; **(G)** *hepcidin a*; **(H)** *ferric-chelate reductase 1a*; **(I)** *ferric-chelate reductase 1b*; **(J)** *ferritin, middle subunit*; **(K)** *apoptosis inducing factor mitochondria associated 2*; **(L)** *BCL2/adenovirus E1B 19 KDa protein-interacting protein 3-like a*.

Forty-two transcripts representing various molecular pathways [innate immune response (i.e. anti-bacterial response, inflammatory response, immune lipid mediators, anti-viral response), cellular immunity (i.e. leukocyte activation and migration, antigen presentation and recognition), and other physiological processes (i.e. oxidative stress response, iron metabolism, apoptosis)] based on GO term enrichment analyses were selected for qPCR confirmation using a complete set of biological replicates collected at 21 DPI (total n = 37; 11 L-SRS, 17 H-SRS, and 9 CON). All of the transcripts evaluated for qPCR confirmation, except for *frrs1b*, agreed with the microarray results in the direction of change (i.e. up- or down-regulation) (**Figures 6**–**8**). In addition, the log_2_ fold-change values calculated from microarray and qPCR data showed a highly significant correlation (R^2^ = 0.8653; *p* < 0.0001), showing excellent overall confirmation of the microarray results by qPCR (**Supplemental Figure S3**).

qPCR results for 14 transcripts involved in the innate immune response are shown in **Figure 6**. Expression levels of *tlr5a*, *campb*, *card9*, *il1b*, *il8a*, *saa5*, *ch25ha* and *alox12* were significantly induced by *P. salmonis* infection in both H-SRS and L-SRS groups (**Figures 6A–C, E–G, I, J**). On the contrary, SRS-induced expression of *c3a* was only significant in the H-SRS group (**Figure 7D**). The expression levels of *gsn*, *pgds*, and three well-known antiviral genes (*rsad2a*, *ifit5*, *mxa*) were repressed by *P. salmonis* infection in both the H-SRS and L-SRS groups except for *rsad2a*, which only showed a significant difference between L-SRS and the control (**Figures 6H, K–N**). All genes in this category, except for the three antiviral genes, exhibited infection level-dependent up-regulation (e.g. *tlr5a*, *campb*, *il8a*, *il1b*, *saa5*, *c3a*) or down-regulation (i.e. *gsn*, *pgds*).

Sixteen transcripts playing roles in the leukocyte activation and function were subjected to qPCR analysis in the current study (**Figure 7**). Genes related to leukocyte activation, differentiation and migration (i.e. *csf1r*, *lgals9c*, *ifng*, *adala*, *mmp13c*, *timp2b*, *aif1*, *bcl10a*, *pstip2a*) were induced in both L-SRS and H-SRS groups, whereas the expression of two other genes (*lgals9b* and *tmod4a*) were repressed compared with the control fish (**Figures 7A–K**). *P. salmonis* infection significantly induced four genes related to antigen presentation (*mhciia1*, *tapbp*, *cd83c*, *b2ma*); however, it suppressed the expression of *tra* (which is involved in antigen recognition) in both L-SRS and H-SRS groups (**Figures 7L–P**). Overall, in this category, 6 genes showed infection level-dependent up-regulation (*ifng*, *timp2b*, *bcl10a*, *pstpip2a*, *b2ma*, *tapbp*), and 1 gene showed infection level-dependent down-regulation (*tmod4a*).

Expression levels of 12 transcripts encoding proteins related to other physiological processes (i.e. redox homeostasis, iron metabolism, and apoptosis) were also evaluated (**Figure 8**). Among the 5 genes related to the oxidative stress response, 2 of them (*ncf2*, *txnb*) had increased expression in fish infected with *P. salmonis* (with *txnb* only significant for L-SRS group), while the other three transcripts (*catc*, *sesn1a*, *selenopb*) showed the opposite regulation (**Figures 8A–E**). Considering iron metabolism-related transcripts, expression levels of *hmox1a*, *hampa*, and *frrs1a* were significantly higher in fish infected with *P. salmonis*, whereas the level of *frrs1b* was not affected (**Figures 8F–I**). In addition, SRS suppressed the transcript expression of *ftm* (**Figure 8J**). Considering apoptotic relevant genes, the expression levels of *aifm2* and *bnip3la* were higher (only for H-SRS) and lower in fish infected with *P. salmonis* compared with the control, respectively (**Figures 8K, L**). Overall, 4 genes (*ncf2*, *hmox1a*, *hampa*, *frrs1a*) exhibited infection level-dependent up-regulation, and 2 genes (*catc*, *sesn1a*) showed infection level-dependent down-regulation among all genes in this category.

### 3.6 Correlation Analyses for the Expression of qPCR-Assessed Transcripts With the Level of *P. salmonis* Infection

Of the 42 qPCR-analyzed transcripts, 25 showed a significant correlation with the level of *P. salmonis* infection (**Table 1**). The RQ values of *hampa*, *frrs1a*, *il8a*, *timp2b*, *campb*, *tlr5a*, *ifng*, *ch25ha*, *il1b*, *pstpip2a*, *bcl10a*, *saa5*, *tapbp*, *card9*, *ncf2*, *hmox1a*, *alox12*, *aifm2* and *c3a* were positively correlated with the infection level, whereas *sesn1a*, *pgds*, *selenopb*, *gsn*, *ftm* and *tra* showed significant negative correlations with the expression level of *P. salmonis ITS*. Interestingly, 13 out of the remaining 17 transcripts (*mhciia1*, *lgals9c*, *aif1*, *adala*, *mmp13c*, *lgals9b*, *csf1r*, *txnb*, *mxa*, *cd83c*, *ifit5*, *bnip3la*, *rsad2a*) that did not have significant correlations with the level of *P. salmonis* infection, still exhibited either an increase or decrease in their expression in infected groups compared with control fish; their expression did not differ between L-SRS and H-SRS groups (**Figures 6**–**8**; **Table 1**).

**Table 1:** Correlation analyses for the expression of qPCR-studied transcripts with the level of *P. salmonis* infection.

Transcript^1^	Direction of change^2^	Pearson r	*p-*value	Theme	Putative Function
***Significant positive correlation***					
*hampa*	Up-regulation	0.841	<0.001	Other physiological processes	Iron metabolism
*frrs1a*	Up-regulation	0.777	<0.001	Other physiological processes	Iron metabolism
*il8a*	Up-regulation	0.775	<0.001	Innate immune response	Inflammatory response
*timp2b*	Up-regulation	0.760	<0.001	Cellular immunity	Leukocyte migration
*campb*	Up-regulation	0.750	<0.001	Innate immune response	Anti-bacterial response
*tlr5a*	Up-regulation	0.703	<0.001	Innate immune response	Anti-bacterial response
*ifng*	Up-regulation	0.689	<0.001	Cellular immunity	Leukocyte activation
*ch25ha*	Up-regulation	0.681	<0.001	Innate immune response	Immune lipid mediators
*il1b*	Up-regulation	0.676	<0.001	Innate immune response	Inflammatory response
*pstpip2a*	Up-regulation	0.646	<0.001	Cellular immunity	Leukocyte migration
*bcl10a*	Up-regulation	0.577	0.001	Cellular immunity	Leukocyte migration
*saa5*	Up-regulation	0.569	0.002	Innate immune response	Inflammatory response
*tapbp*	Up-regulation	0.496	0.007	Cellular immunity	Antigen presentation and recognition
*card9*	Up-regulation	0.467	0.012	Innate immune response	Inflammatory response
*ncf2*	Up-regulation	0.450	0.016	Other physiological processes	Oxidative stress response
*hmox1a*	Up-regulation	0.421	0.026	Other physiological processes	Iron metabolism
*alox12*	Up-regulation	0.413	0.029	Innate immune response	Immune lipid mediators
*aifm2*	Up-regulation	0.385	0.043	Other physiological processes	Apoptosis
*c3a*	Up-regulation	0.378	0.047	Innate immune response	Anti-bacterial response
***Significant negative correlation***					
*sesn1a*	Down-regulation	-0.796	<0.001	Other physiological processes	Oxidative stress response
*pgds*	Down-regulation	-0.640	<0.001	Innate immune response	Immune lipid mediators
*selenopb*	Down-regulation	-0.552	0.002	Other physiological processes	Oxidative stress response
*gsn*	Down-regulation	-0.521	0.004	Innate immune response	Inflammatory response
*ftm*	Down-regulation	-0.441	0.019	Other physiological processes	Iron metabolism
*tra*	Down-regulation	-0.419	0.026	Cellular immunity	Antigen presentation and recognition
***Non-significant correlation***					
*tmod4a*	Down-regulation	-0.351	0.067	Cellular immunity	Leukocyte migration
*catc*	Down-regulation	-0.342	0.075	Other physiological processes	Oxidative stress response
*mhciia1*	Up-regulation	0.298	0.124	Cellular immunity	Antigen presentation and recognition
*lgals9c*	Up-regulation	0.269	0.167	Cellular immunity	Leukocyte activation
*aif1*	Up-regulation	0.249	0.201	Cellular immunity	Leukocyte migration
*b2ma*	Up-regulation	0.244	0.211	Cellular immunity	Antigen presentation and recognition
*adala*	Up-regulation	0.225	0.250	Cellular immunity	Leukocyte activation
*mmp13c*	Up-regulation	0.196	0.318	Cellular immunity	Leukocyte migration
*lgals9b*	Down-regulation	-0.156	0.427	Cellular immunity	Leukocyte activation
*csf1r*	Up-regulation	-0.097	0.624	Cellular immunity	Leukocyte activation
*txnb*	Up-regulation	-0.096	0.627	Other physiological processes	Oxidative stress response
*frrs1b*	–	0.090	0.647	Other physiological processes	Iron metabolism
*mxa*	Down-regulation	-0.069	0.727	Innate immune response	Anti-viral response
*cd83c*	Up-regulation	0.063	0.751	Cellular immunity	Antigen presentation and recognition
*ifit5*	Down-regulation	-0.040	0.839	Innate immune response	Anti-viral response
*bnip3la*	Down-regulation	-0.037	0.850	Other physiological processes	Apoptosis
*rsad2a*	Down-regulation	0.033	0.869	Innate immune response	Anti-viral response

^1^Control fish were excluded from the current analysis.

^2^Direction of transcript expression change as compared to the control groups.

## 4 Discussion

### 4.1 A Low Mortality Model of Piscirickettsiosis in Atlantic Salmon Parr Involving an EM-90-Like Strain but With a Strong Transcriptomic Response in Head Kidney

Improved understanding of the molecular mechanisms involved in the host response to *P. salmonis* will accelerate the development of effective diagnostics, vaccines, therapeutics, and nutritional solutions to combat SRS. The present study used a robust microarray platform, the cGRASP-designed Agilent 44K salmonid oligonucleotide microarray (30), to explore the transcriptomic response of the Atlantic salmon head kidney from a low mortality *P. salmonis* EM-90-like strain infection in freshwater. EM-90-like strains represent an epidemiologically relevant group in the current situation of piscirickettsiosis (e.g. in Chilean salmon aquaculture) (18); however, it has received fewer research efforts compared with that of LF-89-like isolates. Piscirickettsiosis has often been reported in seawater-reared salmonids; the disease in coho salmon and rainbow trout farmed in freshwater has also been documented previously (22, 23, 59). The lower incidence of SRS outbreaks in freshwater may be related to the instability of the bacterium in this environment (59). Nevertheless, experimental infection trials have been successfully demonstrated in freshwater reared coho salmon, Atlantic salmon and rainbow trout with up to 98% mortalities (24–26).

Two recent studies on the development of challenge models involving *P. salmonis* EM-90-like isolates in Atlantic salmon post-smolts showed extremely high mortalities (>95%) in both Trojan and cohabitant groups (20, 21). In contrast, much lower mortalities (28.5-32.7%) were observed in fish injected with an EM-90-like strain in the present study. Although the life stages of salmon in the current study differed from previous challenge models, the lower mortality rate observed here is likely due to the lower amount/concentration of bacterial inoculum given to the fish. A low mortality EM-90-like *P. salmonis* infection model is needed to fill in the gaps in the current knowledge on piscirickettsiosis, and it can be an essential tool to develop and test future preventive measures (e.g. functional feed) against the disease.

The infection prevalence of *P. salmonis* in all fish analyzed was 100% at both 13 and 21 DPI, with the highest transcript level of *P. salmonis ITS* detected at 21 DPI (**Figures 2A, B**). Congruently, the screening of four well-known antibacterial biomarkers (*campb*, *hampa*, *il8a* and *tlr5a*) in salmon head kidney also showed similar immune response dynamics. Although different challenge models (IP vs. cohabitation) were used, our results agree well with a previous study by Rozas-Serri et al. (19), where they found that the expression of many mediators of innate immunity (e.g. *il1b*, *il8*) correlated positively with the bacterial load in the head kidney of Atlantic salmon infected with an EM-90-like isolate. Based on our assessment of immune biomarker gene expression and pathogen levels across all sampling time points, we focused our efforts on profiling the transcriptome response of Atlantic salmon head kidney to *P. salmonis* infection at 21 DPI, a time point that is likely to reveal the highest transcriptome modulation. In addition, all the fish in the current study were infected with the same dose of *P. salmonis* inoculum; however, multivariate statistical analyses (i.e. PCA and clustering analysis) of infected fish at 21 DPI revealed two infection level phenotypes: a lower *P. salmonis* level with lower expression of antibacterial biomarkers (L-SRS); and a higher *P. salmonis* level with higher expression of antibacterial biomarkers (H-SRS) (**Supplemental Figure S1**). These two phenotypes observed in the current study are likely indicative of SRS resistance with H-SRS being more susceptible and L-SRS being less susceptible to *P. salmonis*.

Although several transcriptomic studies have examined the impact of *P. salmonis* infection in Atlantic salmon (4, 10, 11, 17, 60), only one study, carried out by Rozas-Serri et al. (17), focused on an EM-90-like isolate in post-smolts. Despite a relatively low mortality rate observed in the present study, we have identified a much larger set of *P. salmonis*-responsive molecular biomarkers (a total of 3,242 DEPs) in the head kidney of Atlantic salmon compared with the results (298 DEGs in the IP injection model) of Rozas-Serri et al. (17). Although the salmon’s life stage differed between studies, the smaller number of transcripts identified therein may be attributed to the difference in response to the studied time points (5 vs. 21 days). There is a high variation in the transcriptomic response (i.e. total numbers of differentially expressed probes/genes) to infection in Atlantic salmon among studies involving other strains of *P. salmonis* (e.g. LF-89). For instance, the first transcriptome study using a 3.5K GRASP cDNA microarray identified 69 transcripts differentially expressed in response to *P. salmonis* in Atlantic salmon head kidney, following 14 days of infection (4). Pulgar et al. (11), using the same time point (14 DPI) and a 32K cGRASP cDNA microarray, identified approximately 2,500 DEPs between infected and non-infected fish among Atlantic salmon families with varying levels of susceptibility to the infection. In contrast, only 207 *P. salmonis*-responsive transcripts in the head kidney of Atlantic salmon were detected two days post-infection using a different version of the 44K oligo microarray compared with the present study (10). As discussed earlier, this is likely due to a mild transcriptomic modulation during an early stage of *P. salmonis* infection.

To confirm the microarray results, 42 transcripts representing various molecular pathways [innate immune response (e.g. anti-bacterial response, inflammatory response), cellular immunity (e.g. leukocyte activation and migration)], and other physiological processes (e.g. oxidative stress response, iron metabolism] were subjected to qPCR analyses using larger numbers of biological replicates in addition to those that were included in the microarray study. All of the qPCR-analyzed transcripts, except for *frrs1b*, agreed with microarray results in the direction of change and statistical significance in at least one of the comparisons (i.e. L-SRS vs control or H-SRS vs control). A highly significant correlation (R^2^ = 0.8653; *p* < 0.0001) was also obtained between microarray and qPCR data, suggesting an excellent overall confirmation of microarray results by qPCR.

### 4.2 *P. salmonis* Infection Affects Host Innate Immunity

In the present study, we examined the global gene expression response in both lower and higher *P. salmonis* level individuals compared with the time-matched control fish; the identified pathways overlapping between lower and higher infection level groups (L-SRS and H-SRS) may represent the core defense response in Atlantic salmon. The pathway enrichment analysis of SRS-responsive transcripts overlapping between both infection groups (1,470 DEPs) showed a large number of BPs (36.3%) associated with innate immune responses were regulated in response to *P. salmonis* infection (**Figure 4**; **Supplemental Table S4**). Enriched BP terms related to immune response include “positive regulation of NF-kappaB transcription factor activity”, “defense response to bacterium”, “regulation of inflammatory response”, “response to interleukin-1”, and “response to virus and chemotaxis”. Similar to our study, previous transcriptomic analyses of head kidneys in Atlantic salmon infected with *P. salmonis* revealed an activated innate immune defense response mechanism (4, 10, 11, 17).

In the current study, 14 genes directly involved in the innate immune response were analyzed by qPCR (**Figure 6**). *P. salmonis* infection showed up-regulation of *tlr5a*, *campb*, *card9* and *c3a* in both L-SRS and H-SRS groups compared with the control, except for *c3a*, which was only significant in H-SRS group. The flagellin-dependent activation of Tlr5, a pattern-recognition receptor (PRR), has been well recognized in mammals and a similar mechanism has been reported in some teleosts [e.g. rainbow trout and Japanese flounder (*Paralichthys olivaceus*)] (61). However, some recent studies on non-motile bacterial infection models in Atlantic salmon (e.g. *P. salmonis* and *R. salmoninarum*) (17, 31), as well as the current research, suggest that the induction of *tlr5a* in Atlantic salmon may not be flagellin-dependent. Transcripts *campb* and *c3a* encode an antimicrobial peptide and a complement protein, respectively; they are both essential members of humoral components of innate immunity against a large array of pathogens (62). In agreement with our results, the transcription of Atlantic salmon *camp* and *c3* was induced in fish infected with EM-90-like *P. salmonis* isolate (17). Card9 is an adaptor protein that mediates signals from PRRs to activate inflammatory cytokines, playing a key role in the innate immune response to several intracellular pathogens in humans (63). The increased transcription of *campb*, *c3a*, and *card9* in fish infected with *P. salmonis* in the present study indicate stimulation of innate immune response.

The innate immune pathways activated by *P. salmonis* infection modulated the expression of *il1b*, *il8a*, *saa5*, and *gsn*, which are associated with inflammation and acute phase response. Il1b is one of the earliest expressed pro-inflammatory cytokines after activation of host PRRs (64), and enables organisms to respond promptly to an infection by inducing a cascade of reactions leading to inflammation (65). A member of the CXC chemokine family, Il8/Cxcl8, regulated by Il1b, functions as a chemotactic factor by recruiting specific subsets of leukocytes to sites of inflammation and infection (65). As in our study, Rozas-Serri et al. (19) observed up-regulation of both *il1b* and *il8* in the head kidney of Atlantic salmon challenged by LF-89-like and EM-90-like isolates. Saa5 plays a significant role in the acute phase response in animals suffering from infection or injury, and its transcript levels increased dramatically in rainbow trout with *Yersinia ruckeri* infection (66). In mammals, GSN is a crucial regulator of actin filament assembly and disassembly; however, recent evidence from mammals suggests that GSN inhibits the inflammatory and cytokine response induced by LPS and overexpression of GSN decreases inflammation and apoptosis in experimental allergic encephalomyelitis animals (67, 68). Moreover, in agreement with our results, Eslamloo et al. (31) also showed the decreased expression of *gsn* in Atlantic salmon infected with *R. salmoninarum*. The suppressed *gsn* expression may enhance the overall inflammatory response in the *P. salmonis* infected Atlantic salmon.

The present study showed that *P. salmonis* infection modulated transcripts encoding lipid mediators (i.e. *ch25ha*, *alox12*, and *pgds*), which play vital roles in the innate immune response. Ch25h converts cholesterol to oxysterol 25-hydroxycholesterol to maintain cholesterol homeostasis (69). Studies in mammals and fish suggested that Ch25h has additional functions in immunomodulation (e.g. antiviral and antibacterial activities) and can positively and negatively regulate the inflammatory responses (69, 70). Similar to our study, *R. salmoninarum* induced the expression of *ch25ha* in Atlantic salmon head kidney (31). Transcripts *alox12* and *pgds* encode enzymes that act on different polyunsaturated fatty acid substrates to generate bioactive lipid mediators (e.g. eicosanoids) and mediate inflammatory response (71, 72). The observed down-regulation of *pgds* in the head kidney of Atlantic salmon infected with *P. salmonis* in this study agrees well with previous Atlantic salmon infection and immune challenge models (4, 31, 73). These data confirm that lipid mediators could be playing important roles in different infectious diseases in Atlantic salmon.

Three well-known antiviral (or viral-induced) biomarkers studied by qPCR (i.e. *mxa*, *ifit5*, and *rsad2a*) had decreased mRNA expression in fish infected with *P. salmonis* in the present study. Previous studies on sea lice infection models in Atlantic salmon showed similar down-regulation of several antiviral effector genes, including those that were regulated by the interferon pathway (33, 74). Further, sea lice-infected Atlantic salmon that had lower expression of antiviral genes showed higher infectious salmon anemia viral load and mortality (75). It is worth noting that the current microarray analyses also revealed increased expression of additional *mx* paralogues and other transcripts known to be involved in anti-viral response (e.g. *interferon-induced protein 44*, *guanylate-binding protein 1*) (76, 77) upon *P. salmonis* infection (**Supplemental Table S3**). Therefore, it is possible that some aspects of anti-viral immunity were altered, which could influence their susceptibility to viral infection.

### 4.3 *P. salmonis* Infection Affects Host Cellular Immunity

The present study showed that SRS influenced molecular pathways (e.g. “leukocyte migration”, “leukocyte activation”, “CD4-positive alpha-beta T cell activation”, “antigen processing and presentation of exogenous antigen”) involved in both innate and adaptive cellular immunity. More than one-third of over-represented BPs from the overlapping SRS-responsive transcript list (1,470 DEPs) were related to adaptive immunity. This large number of enriched adaptive immune response relevant BPs is somewhat unexpected since relatively low numbers of *P. salmonis*-responsive transcripts associated with adaptive immune response were identified by other relevant studies (4, 10, 11, 17). Nevertheless, Atlantic salmon infected with live *Renibacterium salmoninarum* (causative agent of Bacterial Kidney Disease) also revealed an extensive amount of adaptive immune processes being regulated (31).

Sixteen transcripts studied by qPCR in the current investigation play essential roles in leukocyte activation, migration, and function, and their mRNA levels in Atlantic salmon head kidney were significantly modulated by *P. salmonis* infection (**Figure 7**). For instance, *P. salmonis* induced four transcripts (i.e. *csf1r*, *lgals9c*, *ifng*, *adala*) and suppressed one transcript (i.e. *lgals9b*) that play putative roles in macrophage and/or lymphocyte activation. Csf1r acts as the receptor for colony stimulating factor 1, which regulates the production, differentiation, and function of monocytes/macrophages (78). In grass carp (*Ctenopharyngodon idellus*), Csf1r was identified as a specific surface marker of monocytes/macrophages, and its transcript level in various tissues was elevated in fish infected with *Aeromonas hydrophila* (79). Mammalian LGALS9 induces antibacterial activity in macrophages infected with *Mycobacterium tuberculosis* through macrophage activation and IL1B secretion (80), consistent with the up-regulation of one *lgals9* paralogue (i.e. *lgals9c*) in salmon infected with *P. salmonis* found in the present study. The opposite transcript expression change of *lgals9b* observed here suggests that these paralogues have undergone regulatory and potentially functional divergence. IFNG or type II interferon is a cytokine that exerts regulatory roles in both innate and adaptive immunity, including activating macrophages, enhancing antigen presentation and promoting T cell differentiation and activation (10, 31, 64, 81). Adal, an enzyme involved in the salvage of purine nucleotides, also plays an important role in regulating T cell activation and differentiation (82, 83).

The present qPCR study showed that *P. salmonis* infection modulated transcripts that play key roles in immune cell migration and actin/cytoskeleton reorganization (*mmp13c*, *timp2b*, *aif1*, *bcl10a*, *pstpip2a*, *tmod4a*) (**Figure 7**). Matrix metalloproteinases (MMPs) are a family of zinc-containing proteolytic enzymes that exert multiple roles in the immune response to infection, including facilitating leucocyte recruitment and migration, modulating cytokine and chemokine activity, and extracellular matrix remodeling (84). In the zebrafish heart regeneration model, the inhibition of both Mmp9 and Mmp13 resulted in impaired tissue regeneration and leukocyte recruitment (85). However, excess Mmp activity following infection may lead to immunopathology favouring the pathogen, agreeing with the up-regulation of transcript encoding Timp2b, a regulatory inhibitor of Mmps, in *P. salmonis*-infected salmon. The cytoskeleton and its components (e.g. different actins), well known for their roles in cell division, shape and movement, have essential functions in innate immunity and cellular self-defense (17, 60, 86). In mammals, transcripts encoding AIF1, PSTPIP2, and BCL10 positively regulate actin polymerization and/or filopodia formation, while Tmod4 prevents actin filaments from elongation (87–92). The activated actin polymerization is likely to contribute to the promotion of immune cell mobility and migration in *P. salmonis* infected animals (11). The transcript expression of *tmod4* in Atlantic salmon was also suppressed by *R. salmoninarum* (31). Future studies are needed to better understand the function of cytoskeletal remodeling during piscirickettsiosis. Part of the observed responses could contribute to phagocytosis, which may eliminate *P. salmonis* bacteria. At the same time, intracellular bacterial pathogens such as *P. salmonis* and *R. salmoninarum* likely need to modulate cytoskeleton rearrangements to favor their replication in host cells (60, 93).

The activation of cytotoxic T lymphocytes by recognition of antigenic peptides presented on major histocompatibility complex (MHC) molecules associated with antigen-presenting cells (APCs) is a critical process in the adaptive immunity of vertebrates (94, 95). As shown by the current qPCR analyses, transcripts involved in antigen processing, presentation, and recognition (i.e. *b2ma*, *tapbp*, *mhciia1*, *cd83c*, *tra*) were modulated by *P. salmonis* infection (**Figure 7**). B2m is a component of MHC class I molecules, which bind and present endogenously derived peptides to CD8^+^ T-cells (95). Tapbp is an MHC class I antigen-processing molecule present in the endoplasmic reticulum that helps the stabilization of a multimeric peptide-loading complex (94). Unlike MHC class I, the MHC class II molecules primarily reside on the surface of professional APCs such as dendritic cells which present the antigens derived from extracellular proteins to CD4^+^ T-cells (95, 96). In mammals, CD83 is a member of the immunoglobulin (Ig) superfamily and a surface marker on mature dendritic cells (97). In addition to the roles that CD83 plays in lymphocyte development, a previous study revealed that CD83 influences cell-surface MHC class II expression on B cells and other APCs in mice, therefore affecting antigen presentation (98). The increased transcript expression of genes involved in both MHC class I and II in the present study suggests the importance of antigen processing and presentation pathways in the Atlantic salmon response to SRS. Interestingly, the current microarray study identified 69 down-regulated probes related to T-cell receptors [63 for T-cell receptor alpha (*tra*) and 6 for T-cell receptor beta] in fish infected with *P. salmonis* (**Supplemental Table S3**) with the suppression of *tra* confirmed by qPCR in both L-SRS and H-SRS fish. Tra is a protein complex found on the surface of T-cells responsible for recognizing fragments of antigen presented by MHC molecules (99). In agreement with our results, previous studies also reported the down-regulation of *tra* in *P. salmonis*-infected Atlantic salmon head kidney (4, 10). CD274 could also play a role in the suspected suppression of T-cell activity in the current study. This inhibitory receptor ligand, induced in both L-SRS and H-SRS fish (**Supplemental Table S3**), binds to the receptor PD-1, commonly expressed on T-cells, thereby blocking T-cell activation (100).

### 4.4 *P. salmonis* Infection Affects Other Physiological Processes

Apart from the regulation of immune pathways and processes, the current microarray data also showed genes involved in other physiological processes [e.g. “response to reactive oxygen species” (**Figure 4**), “transition metal ion transport” and “programmed cell death” (**Figure 5B**)] were affected in *P. salmonis*-infected Atlantic salmon. We selected 12 microarray-identified genes related to these processes for qPCR confirmation (**Figure 8**). It is well-known that reactive oxygen species (ROS) play a dual role in pathogenic infections (101). ROS (i.e. free radicals) can protect the host from invading pathogens. However, their overproduction can cause oxidative stress, resulting in tissue damage. The up-regulation of *ncf2*, part of the NADPH oxidase components (102), suggests the elevated production of free radicals in response to *P. salmonis* infection. This result is consistent with the up-regulation of NADPH oxidase in the liver of Atlantic salmon infected with *P. salmonis* found in a previous study (10). Interestingly, the transcript encoding for the protein Txn, a critical antioxidant enzyme, was up-regulated only in L-SRS group. The up-regulation of *txnb* in L-SRS group may be a protective response to oxidative stress resulting from *P. salmonis* infection. In the current study, *P. salmonis* infection also suppressed transcripts encoding enzymes that are important in the antioxidant defense system (e.g. *catc*, *sesn1a*, and *selenopb*). Similar results for *selenopb* transcript expression was reported in a previous study on Atlantic salmon head kidney infected with *P. salmonis* (4). Moreover, Rozas-Serri et al. (17) found that more than 10 genes associated with the anti-oxidative response were down-regulated in the head kidney of *P. salmonis* infected Atlantic salmon. These results suggest that *P. salmonis* infection modulates the host antioxidant system, which may result in severe tissue damage.

Iron is a crucial nutrient for the survival of bacteria and a key regulator of the host-pathogen interaction (103). Of the iron metabolism relevant genes studied by qPCR, our results showed that *P. salmonis* infection up-regulated the expression of *hampa*, *frrs1a* and *hmox1a*, and down-regulated the expression of *ftm* in both the L-SRS and H-SRS groups. It is well-known that HAMP exhibits antimicrobial activity against bacteria and fungi (104). It also plays a key role in the maintenance of iron homeostasis and the negative regulation of iron efflux in macrophages (105, 106). FRRS1 (also referred to as SDR2) reduces ferric to ferrous iron before its transport from the endosome to the cytoplasm (107). Iron withholding response or nutritional immunity is a process initiated by infection-induced inflammation that aims to deprive invading pathogens from circulating iron; the host sequesters iron within intracellular pools which is thought to starve pathogens of this essential nutrient further limiting disease progression (12, 13). The activation of nutritional immunity was evidenced by the up-regulation of *hampa*, *frrs1a*, *haptoglobin* and *hemopexin* (microarray-identified; **Supplemental Table S3**) that increase iron intracellular storage, while the slight down-regulation of *ftm* suggests that the response needs to be finely tuned. Pulgar et al. (11) found that significant up-regulation of *hamp* was only shown in Atlantic salmon families with high susceptibility to *P. salmonis* but not in the low susceptibility families. Given that *P. salmonis* is a facultative intracellular bacterium, iron withholding response may be more detrimental for the host and more beneficial to *P. salmonis* once established inside the cells. Recently, several groups have successfully treated *P. salmonis* infection with iron chelators (12, 16) – induction of the nutritional immunity (iron withholding response) and limiting access of iron to bacteria under this condition appears to be a viable disease control strategy. The lack of sufficient amounts of extracellular iron resulted from iron withholding response likely had adverse consequences for the differentiation of red blood cells in the head kidney, which is considered to be the primary site of erythropoiesis in teleost fish (108). This was evidenced by the decrease in the expression of erythrocyte-specific genes (e.g. *spectrin beta chain* and *hemoglobin*) (109), and a gene governing erythropoiesis (*erythropoietin receptor*) (110) in *P. salmonis* infected fish as shown by the current microarray analyses (**Supplemental Table S3**). The increased transcript expression of *hmox1*, an essential enzyme in the breakdown of heme originating from degraded senescent erythrocytes and heme-proteins, producing equal amounts of iron, carbon monoxide and biliverdin (111), suggested that the heme metabolism was also affected by SRS.

Our results suggest that *P. salmonis* infection affects the host redox status, which may influence cell death and necrosis. Previous studies have shown that *P. salmonis* modulates cellular apoptosis to enable their replication and survival within the host cells (4, 10, 17, 60). Of the apoptosis-related genes studied by qPCR, our results revealed that *P. salmonis* infection up-regulated the expression of *aifm2* in H-SRS and down-regulated the expression of *bnip3la* in both the L-SRS and H-SRS groups. Human AIFM2 (also referred to as AMID) has a pro-apoptotic function and is a p53 target to promote caspase-independent cell death (112). Bnip3 is another pro-apoptotic gene that belongs to the Bcl-2 protein family (113). Moraleda et al. (60) also showed that *bnip3* was down-regulated in Atlantic salmon during SRS infection, and it was positively correlated with resistance to SRS. Taken together, these results suggest that the host may stimulate apoptotic pathways as a means to control pathogen dissemination, while *P. salmonis* needs to ensure the maintenance of host cells in which it resides.

### 4.5 Genes and Their Expression That Might Be Associated With SRS Resistance

As mentioned earlier, the present study also aimed to explore the transcriptomic differences between individuals having higher and lower pathogen loads (L-SRS vs H-SRS), which could potentially provide insight into disease resistance mechanisms (11, 27, 28). At the pathway level (i.e. GO term enrichment), we analyzed two groups of DEPs between infected and non-infected fish. The first group included 166 probes that were significantly modulated only in the L-SRS group, and the second group had 1606 probes that were significantly modulated only in the H-SRS group. Although a relatively small number of DEPs were exclusive to the L-SRS group, the enriched GO terms were primarily associated with the immune response (36.8%; e.g. “response to type I interferon”, “regulation of complement activation”) and the adaptive immune response (36.8%; e.g. “alpha-beta T cell activation” and “cellular response to interferon-gamma”), suggesting the importance of these pathways (e.g. IFN-mediated) for the positive outcome of *P. salmonis* infection. The enriched GO terms associated with 1606 DEPs responsive only in the H-SRS group were similar to the enriched terms related to the overlapping SRS-responsive genes in terms of number and diversity. It included not only pathways related to immune responses but also pathways representing more general physiological processes such as development, metabolic process, and response to stress. This observation suggests that the transcriptional response to *P. salmonis* in the H-SRS group was more pronounced compared with the L-SRS group. In a previous *P. salmonis* challenge study in Atlantic salmon, resistant individuals had lower levels of bacterial load and higher expression of *c-type lysozyme* in head kidney, while susceptible fish presented with higher levels of bacterial load and higher expression of pro-inflammatory genes and genes involved in acute phase response (27). As indicated by several studies of bacterial (15, 114) and viral diseases (28, 29, 115) in salmonids, exaggerated innate immune responses are often linked to increase in pathology and other adverse outcomes.

At the gene level, we correlated the levels of qPCR-analyzed transcripts with the *P. salmonis* level. Among them, 19 transcripts had a significant positive correlation with the *P. salmonis* level, and many of them are related to iron metabolism (e.g. *hampa*, *frrs1a*), inflammatory response (e.g. *il8a*, *saa5*), antibacterial response (e.g. *campb*, *c3a*) and leukocyte function (e.g. *ifng*, *bcl10a*). There were 6 transcripts that had a significantly negative correlation with the *P. salmonis* level; these included genes involved in oxidative stress response (i.e. *sesn1a*, *selenopb*). As discussed earlier, some of these infection level-dependent responses (e.g. antibacterial response) are beneficial in eliminating the pathogen, while others (e.g. inflammatory response, iron withholding response) may be detrimental to the host. The significant correlations of these transcripts with the infection level suggest they could be suitable biomarkers for the assessment of infection level-dependent SRS responses in Atlantic salmon. It is worth mentioning that 13 qPCR-analyzed transcripts had a similar magnitude of increase or decrease in expression between the L-SRS and H-SRS groups. Among these were genes involved in cellular immunity [leukocyte function (e.g. *lgals9b*, *lgals9c*, *aif1*, *adala*, *mmp13c*, *csf1r*), antigen presentation and recognition (e.g. *mhciia1*, *cd83c*)], anti-viral response (e.g. *mxa*, *ifit5*, *rsad2a*), apoptosis (*bnip3la*), and oxidative stress response (*txnb*). These results suggest that the L-SRS fish can effectively modulate those important immune pathways, which may be key for SRS resistance.

## 5 Conclusion

In summary, the time-course analyses of pathogen load and four biomarkers of innate immunity in the head kidney of Atlantic salmon parr challenged with EM-90-like *P. salmonis* bacterium revealed two groups of fish with different infection levels at 21 DPI. Although the challenge resulted in a relatively low mortality rate, transcriptome profiling of infected fish revealed that piscirickettsiosis affected a great number of genes and pathways, particularly in the high infection group (i.e. H-SRS). High expression levels of genes involved in innate and adaptive immune processes were observed, while a relatively mild regulation of genes governing general physiological processes (e.g. development and metabolism) was seen in response to the *P. salmonis* infection. Furthermore, the comparison of individuals with differing levels of infection (H-SRS vs L-SRS) generated insights into the biological processes possibly involved in natural resistance. A more pronounced immune response against infection at a late stage observed in H-SRS fish suggests that at least part of these responses was exaggerated and not protective. It is however also possible that the H-SRS fish need to maintain a stronger immune response in order to deal with the high pathogen load and prevent further bacterial growth. To help fully elucidate mechanisms responsible for different infection phenotypes (L-SRS and H-SRS) and protection against *P. salmonis* infection, examination of earlier time points (e.g. 2 DPI) and other relevant tissues (e.g. spleen and liver) are necessary. It should be noted that aspects such as route of infection, infection level, and patterns of disease spread during natural SRS outbreaks may differ from the IP injection model. Finally, this study demonstrated a low mortality EM-90-like *P. salmonis* infection model and qPCR-validated many SRS-responsive molecular biomarkers, which are valuable tools for future research (e.g. evaluation of novel functional feeds that require low mortality outcomes) aimed at improving farmed Atlantic salmon resistance to SRS.

## Data Availability Statement

The whole microarray dataset of 20,701 probes is available in NCBI's Gene Expression Omnibus database (https://www.ncbi.nlm.nih.gov/geo/) under the accession GSE178327.

## Ethics Statement

All procedures in the fish infection trial were conducted following the guidelines of the Canadian Council on Animal Care (CCAC, 2005).

## Author Contributions

XX took a lead role in TaqMan assays, sample selection, microarray experimental design, microarray analyses, qPCR validation design, data analyses, data interpretation, and the writing of the manuscript draft. AC-S helped with statistical analyses and enrichment analyses, and reviewed the manuscript. JRH conducted qPCR analysis of pathogen load and candidate host immune biomarkers, and reviewed the manuscript. UN helped with microarray hybridization and data analyses, and reviewed the manuscript. SK assisted with the annotation of the microarray results. EJ managed the experimental design, infection trial and samplings, and took a part in manuscript preparation. SS took a part in data interpretation and manuscript preparation. CH managed the experimental design, infection trial and samplings. JS took a part in data interpretation, and reviewed the manuscript. RGT took a part in funding acquisition and data interpretation. MLR was involved in experimental design, sample selection, microarray experimental design, data analyses and data interpretation, and took an active role in manuscript writing. All authors read and approved the final manuscript.

## Funding

This study was part of two Genomic Applications Partnership Program (GAPP) projects [the Biomarker Platform for Commercial Aquaculture Feed Development project (#6604) and the Integrated Pathogen Management of Co-infection in Atlantic salmon (IPMC) project (#6607)], funded by the Government of Canada through Genome Canada and Genome Atlantic, and EWOS Innovation (now part of Cargill, Incorporated). The IPMC project was also funded by InnovateNL (Government of Newfoundland and Labrador, Department of Tourism, Culture, Industry and Innovation; Leverage R&D award #5401-1019-108). Funding was also provided by a Natural Sciences and Engineering Research Council of Canada (NSERC) Discovery Grant (2020-04519) to MLR. XX is supported by a Postgraduate Scholarship-Doctoral (PGS D) from NSERC and a Memorial University of Newfoundland SGS fellowship. Three qPCR assays (*il1b*, *il8a*, and *ch25ha*) for *P. salmonis*-responsive biomarkers were initially developed within a project titled “Genomic studies of salmon macrophage responses to *Piscirickettsia salmonis*”, which was led by Dr. Tiago S. Hori and funded *via* a Terra Nova Young Innovator Award to MLR.

## Conflict of Interest

Authors EJ, SS, CH and RGT were employed by company Cargill Incorporated.

The remaining authors declare that the research was conducted in the absence of any commercial or financial relationships that could be construed as a potential conflict of interest.

The authors declare that this study received funding from EWOS Innovation (now part of Cargill, Incorporated). Authors EJ, SS, CH, and RGT, in the representation of Cargill, Incorporated, participated in the design of infection trial, sample collection, data interpretation, and manuscript preparation. However, they had no role in the design, data collection and analysis of gene expression experiments, and the decision to submit the manuscript for publication.

## Publisher’s Note

All claims expressed in this article are solely those of the authors and do not necessarily represent those of their affiliated organizations, or those of the publisher, the editors and the reviewers. Any product that may be evaluated in this article, or claim that may be made by its manufacturer, is not guaranteed or endorsed by the publisher.
