# Supplementary material for: Transcriptome Profiling of Atlantic Salmon (Salmo salar) Parr With Higher and Lower Pathogen Loads Following Piscirickettsia salmonis Infection
Source: Front Immunol. 2021 Dec 31;12:789465. doi: 10.3389/fimmu.2021.789465 (PMC8758579; doi:10.3389/fimmu.2021.789465)
Supplement: Supplementary file 1 [file DataSheet_1.zip › Supplemental files/Supplemental Figures S1-3_Xue.docx]

**
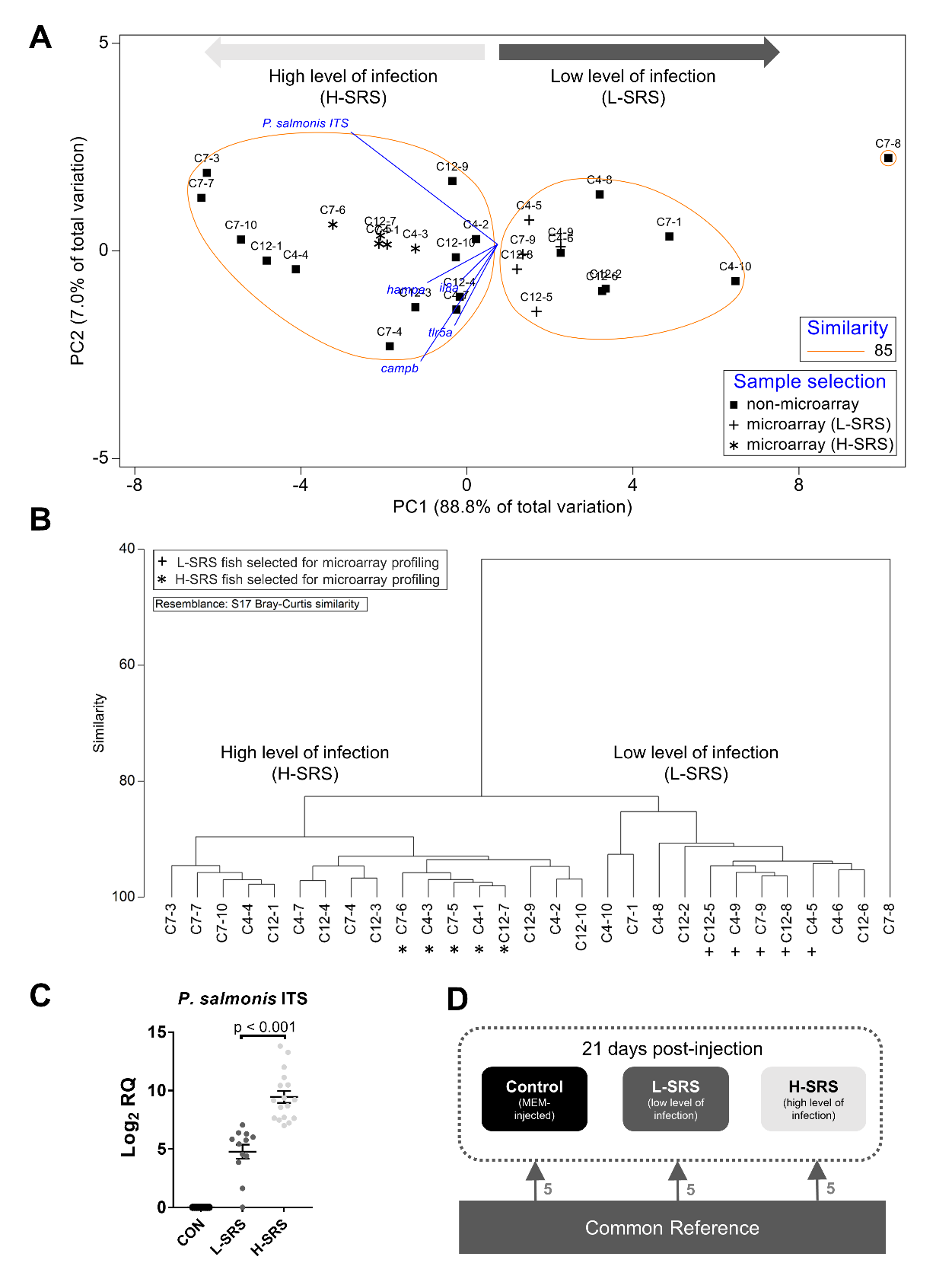
**

**Supplemental Figure S1.** Classification of infection level phenotypes by multivariate statistical analyses and microarray experimental design. (**A**) principal component analysis (PCA) and (**B**) hierarchical clustering of the expression levels of *P. salmonis* ITS and anti-bacterial biomarkers (*campb*, *hampa*, *il8a* and *tlr5a*) of 21 DPI individuals. Hierarchical clustering analyses performed using Pearson’s correlation resemblance matrices (PRIMER, Version 6.1.15, Ivybridge, UK). Two infection level phenotypes were identified except fish C7-8: one group of fish with higher levels of the *P. salmonis* ITS and the anti-bacterial biomarkers (H-SRS) and another group of fish with lower levels of the *P. salmonis* ITS and the anti-bacterial biomarkers (L-SRS). (**C**) Levels of *P. salmonis* ITS in fish classified as L-SRS and H-SRS compared to the control fish (CON). (**D**) microarray experimental design. Five fish from each group were selected and used for the transcriptome analyses.

**
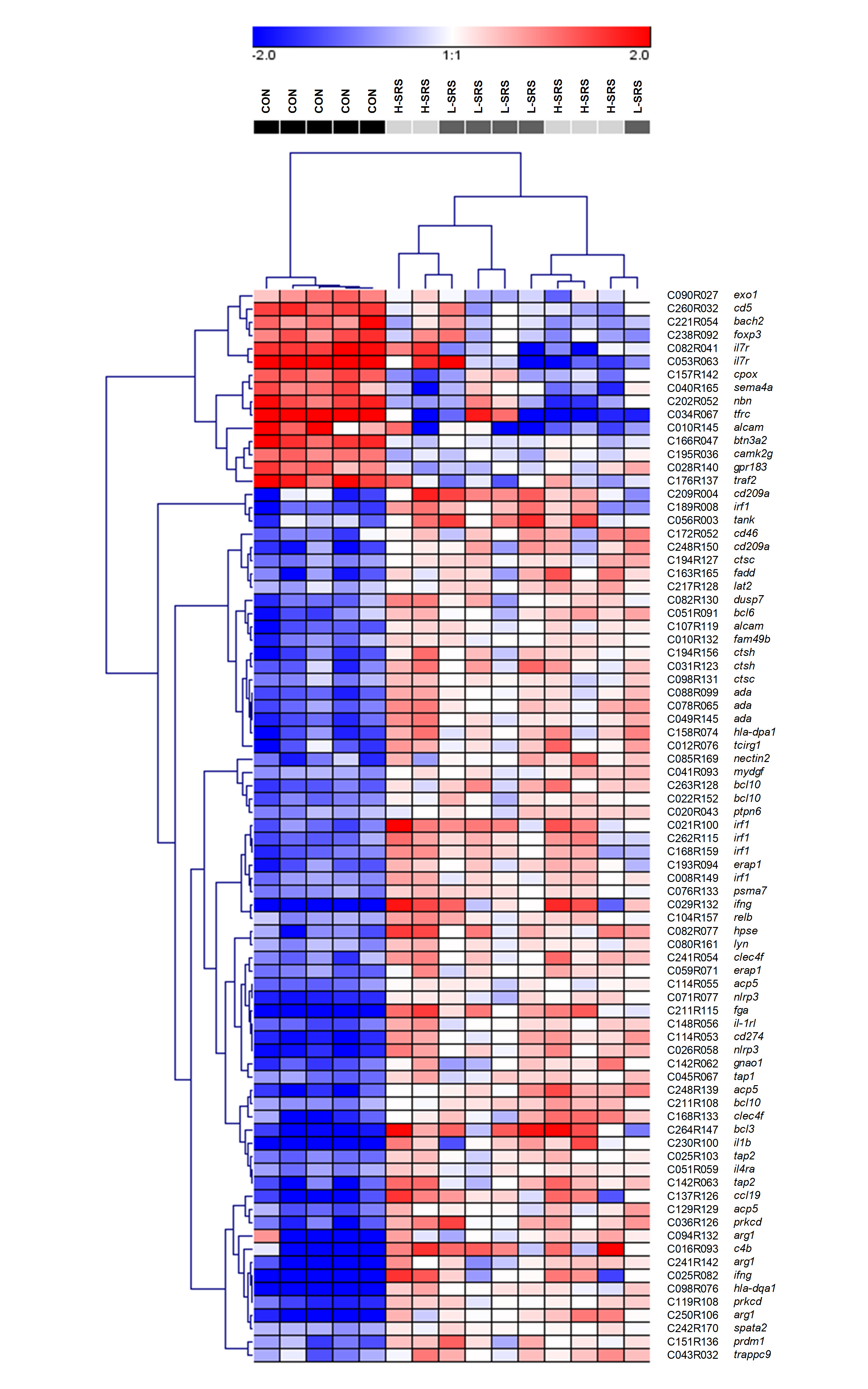
**

**Supplemental Figure S2.** Hierarchical clustering analysis for a subset of SRS-responsive genes involved in adaptive immunity. All microarray analyzed samples were clustered based on probes having the associated GO term “adaptive immune response”.

**Supplemental Figure S3.** Scatterplot of gene expression fold-change values (log_2_) between treatments calculated from the microarray log_2_ ratios and qPCR relative quantity (RQ) values. Each dot represents the fold-change of treatment comparison (L-SRS vs. control or H-SRS vs. control) for a given gene.
